# A widely distributed phosphate-insensitive phosphatase presents a route for rapid organophosphorus remineralization in the biosphere

**DOI:** 10.1073/pnas.2118122119

**Published:** 2022-01-26

**Authors:** Ian D.E.A. Lidbury, David J. Scanlan, Andrew R. J. Murphy, Joseph A. Christie-Oleza, Maria M. Aguilo-Ferretjans, Andrew Hitchcock, Tim J. Daniell

**Affiliations:** ^a^Plants, Photosynthesis and Soil, School of Biosciences, University of Sheffield, Sheffield S10 2TN, United Kingdom;; ^b^School of Life Sciences, University of Warwick, Coventry CV4 7AL, United Kingdom;; ^c^Department of Biology, University of the Balearic Islands, Palma 07122, Spain

**Keywords:** organic phosphorus, flavobacteria, *Bacteroidetes*, phosphatase, PafA

## Abstract

The regeneration of bioavailable phosphate from immobilized organophosphorus represents a key process in the global phosphorus cycle and is facilitated by enzymes known as phosphatases. Most bacteria possess at least one of three phosphatases with broad substrate specificity, known as PhoA, PhoX, and PhoD, whose activity is optimal under alkaline conditions. The production and activity of these phosphatases is repressed by phosphate availability. Therefore, they are only fully functional when bacteria experience phosphorus-limiting growth conditions. Here, we reveal a previously overlooked phosphate-insensitive phosphatase, PafA, prevalent in *Bacteroidetes*, which is highly abundant in nature and represents a major route for the regeneration of environmental phosphate. Using the enzyme from *Flavobacterium johnsoniae*, we show that PafA is highly active toward phosphomonoesters, is fully functional in the presence of excess phosphate, and is essential for growth on phosphorylated carbohydrates as a sole carbon source. These distinct properties of PafA may expand the metabolic niche of *Bacteroidetes* by enabling the utilization of abundant organophosphorus substrates as C and P sources, providing a competitive advantage when inhabiting zones of high microbial activity and nutrient demand. PafA, which is constitutively synthesized by soil and marine flavobacteria, rapidly remineralizes phosphomonoesters releasing bioavailable phosphate that can be acquired by neighboring cells. The *pafA* gene is highly diverse in plant rhizospheres and is abundant in the global ocean, where it is expressed independently of phosphate availability. PafA therefore represents an important enzyme in the context of global biogeochemical cycling and has potential applications in sustainable agriculture.

## Significance

At several locations across the globe, terrestrial and marine primary production, which underpin global food security, biodiversity, and climate regulation, are limited by inorganic phosphate availability. A major fraction of the total phosphorus pool exists in organic form, requiring mineralization to phosphate by enzymes known as phosphatases prior to incorporation into cellular biomolecules. Phosphatases are typically synthesized in response to phosphate depletion, assisting with phosphorus acquisition. Here, we reveal that a unique bacterial phosphatase, PafA, is widely distributed in the biosphere and has a distinct functional role in carbon acquisition, releasing phosphate as a by-product. PafA, therefore, represents an overlooked mechanism in the global phosphorus cycle and a hitherto cryptic route for the regeneration of bioavailable phosphorus in nature.

Both terrestrial and aquatic biological production are regulated by the availability of phosphorus (P), with consequences for global food production, biodiversity, and drawdown of atmospheric CO_2_ (1, 2). The global P cycle therefore plays a vital role in sustaining human existence, both presently and into the future, when P limitation is predicted to constrain the stimulation of land plant biomass in response to elevated atmospheric CO_2_ (2, 3). Likewise, in many regions of the global ocean, primary production by marine phytoplankton is limited by low P availability, predominantly inorganic phosphate (Pi) (4). In terrestrial and marine biomes, a large fraction of the total P pool consists of organic compounds such as phosphonates, phosphomonoesters, phosphodiesters, and phosphotriesters (4–7). Remineralization of organophosphorus into Pi by either a primary producer or its associated microorganisms enhances production through alleviation of P starvation (4, 8, 9). However, knowledge of the environmental distribution of organophosphorus mineralizing enzymes and the relative contribution of distinct microbial taxa toward this process is limited (4, 10). This reduces our ability to predict the influence of anthropogenic-driven global change on marine and soil P cycling, its interaction with the global carbon (C) cycle, and the development of sustainable agricultural tools promoting more efficient crop and animal production.

Bacteria, like all organisms on Earth, require P for survival, growth, and reproduction. The preferred source of exogenous P for bacteria is Pi. However, in the environment, Pi is frequently found at very low or growth-limiting concentrations. Common to bacteria is the ability to overcome environmental Pi scarcity through expression of various genes encoding Pi-stress response proteins (11). This includes the Pi-dependent production of periplasmic or outer membrane–bound enzymes called phosphatases, which cleave the Pi moiety from various organophosphorus compounds. Typically, phosphatases target either organic phosphomonoesters, such as sugar phosphates, or phosphodiesters, such as DNA and phospholipids. Phosphatases can be separated into different classes based on their substrate range (promiscuous or specific), substrate preference (phosphomonoesterases [PMEs], phosphodiesterases [PDEs], or phosphotriesterases) and their pH optimum (acid or alkaline) (10–13). The most common class of bacterial phosphatases are promiscuous alkaline PMEs, which can be further separated into three major families, PhoA, PhoD, and PhoX (14–16). In addition, there is a growing body of evidence that these alkaline PMEs are also active against phosphodiesters and phosphotriesters, broadening the role of these enzymes (17, 18). The unifying function of these enzymes is the production of Pi during times of Pi depletion; consequently, their regulation and enzyme activity are inhibited by ambient concentrations of Pi (19–22). A unique but understudied fourth class of promiscuous bacterial alkaline PME also exists (23) and was recently shown to be highly prevalent in the genomes of *Bacteroidetes* (24). Unlike PhoA, PhoD, and PhoX, this enzyme, referred to as PafA, is not repressed by Pi at either the regulatory (expression of *pafA*) or enzyme activity level (18, 23). Therefore, although the metabolic role of PafA is unknown, regulatory and biochemical data suggests its function is broader than scavenging Pi during times of Pi limitation.

In marine, gut, and soil microbiomes, members of the phylum *Bacteroidetes* are major degraders of plant and algal glycans, occupying a functional niche focused on the degradation of high-molecular-weight (HMW) organic polymers (25–27). Recently, plant-associated *Bacteroidetes* have been shown to play a major role in suppressing plant disease, and there is a growing interest in their ability to augment plant nutrition (24, 28–30). A defining genomic signature of *Bacteroidetes* is their possession of specialized outer membrane transporters, commonly referred to as SusCD (the archetypal transporter is the C and D subunits of the Starch Utilization System), that facilitate the uptake of large polymers for nutrition (31). This is coincident with the apparent lack of adenosine triphosphate (ATP)-binding cassette (ABC) transporters required for the active transport of smaller molecules. Therefore, in addition to specializing in the degradation of HMW polymers, *Bacteroidetes* must possess fundamentally different molecular mechanisms to capture nutrients in comparison to other bacterial taxa. Members of the Bacteroidetes phylum, predominantly *Flavobacteraceae* and *Sphingobacteraceae*, are heavily enriched in the plant microbiome relative to surrounding bulk soil communities, representing 5 to 65% the total microbial community (25, 32–35). Thus, the *Bacteroidetes* phylum must be competitive for various growth-limiting nutrients, such as C, N, and P, despite an apparent lack of transport systems required for nutrient acquisition.

Recently, we discovered that plant-associated *Flavobacterium* spp. possess remarkable potential to mobilize organic P (24). This included the synthesis of numerous PMEs and PDEs and the induction of P-regulated gene clusters, some of which encode SusCD-like transporters, which we termed Phosphate Utilization Systems (PusCD). In addition, numerous *Flavobacterium* spp., especially plant-associated strains, lack a high-affinity phosphate ABC transporter, strengthening the hypothesis that this phylum has divergent mechanisms for nutrient acquisition when exogenous Pi concentrations are very low. Another key characteristic of soil *Bacteroidetes* was the constitutive production of PME activity in the presence of excess exogenous Pi. Through protein fractionation using *Flavobacterium johnsoniae*, PafA was identified as the likely candidate for this unusual PME activity (24), which agrees with previous enzyme kinetic studies on this family of PME (23).

Here, we utilized bacterial genetics to identify the contribution of various predicted PMEs using *F. johnsoniae* as the model. We also tested the hypothesis that the Pi-irrepressible PME, PafA, has a primary role other than Pi scavenging under P-limiting growth conditions. We demonstrate that PafA identified in soil *Bacteroidetes* is highly active toward phosphomonoesters and is essential for growth on sugar phosphate phosphomonoesters as a sole C and energy source. Analysis of metagenomic and metatranscriptomic datasets revealed that *pafA* is abundant in nature and actively transcribed across most regions of the global ocean.

## Results

### *Flavobacterium* PafA Is a Highly Active Phosphomonoesterase.

**Fig. 1.**
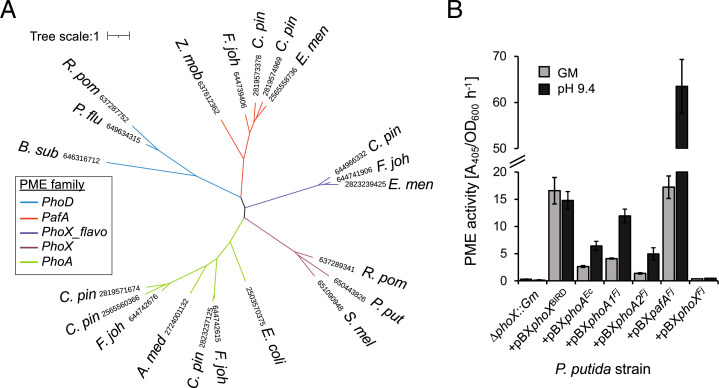
Phylogeny and PME activity for members of the major bacterial alkaline phosphatase families. (*A*) Unrooted phylogenetic tree comparing PhoD, PhoX, and PhoA with PafA homologs. Tree topology and branch lengths were calculated by maximum likelihood using the Blosum62+F+G4 model of evolution for amino acid sequences based on 875 sites (595 parsimony informative) in IQ-TREE software. A consensus tree was generated using 1,000 bootstraps. Numbers indicate IMG accessions. Abbreviations: *F. joh*, *Flavobacterium johnsoniae*; *C. pin*, *Chitinophaga pinensis*; *E. men*, *Elizabethkingia meningoseptica*; *R. pom*, *Ruegeria pomeroyi*; *B. sub*, *Bacillus subtilis*; *P. flu*, *Pseudomonas fluorescens*; *P. put*, *Pseudomonas putida*; *Z. mob*, *Zymomonas mobilis*; *S. mel, Sinorhizobium meliloti*; *A. med*, *Alteromonas mediterranea*. (*B*) PME activity of a *P. putida* Δ*phoX* mutant (Δ*phoX*::*Gm*) complemented with PMEs from *P. putida* BIRD (+pBX*phoX*^BIRD^), *E. coli* (+pBX*phoA^Ec^*), and *F. johnsoniae* (+pBX*pafA^Fj^*, +pBX*phoA1^Fj^*, +pBX*phoA2^Fj^*, and +pBX*phoX^Fj^*) was recorded in cell cultures grown overnight (*n* = 3) in minimal Pi-deplete medium. PME activity was obtained through addition of the artificial substrate *p*NPP (10 mM) under two conditions: the original growth medium (GM) or by resuspending cells in a buffer adjusted to pH 9.4. Values presented are the mean of biological triplicates, and error bars denote SD.

*F. johnsoniae* DSM2064 (hereafter referred to as DSM2064) produces four alkaline PMEs (encoded by *fjoh_0023*, *fjoh_3187*, *fjoh_3249*, and *fjoh_2478*) that are related to previously identified PMEs (Fig. 1*A* and Table 1) (24). The PME encoded by *fjoh_2478* (hereafter PhoX*^Fj^*) is a predicted lipoprotein distantly related to PhoX, which was abundantly secreted in response to Pi depletion. Two periplasmic PMEs, encoded by *fjoh_3249* (hereafter PhoA1*^Fj^*) and *fjoh_3187* (hereafter PhoA2*^Fj^*), are related to PhoA. The fourth, encoded by *fjoh_0023* (hereafter PafA*^Fj^*), is related to a Pi-irrepressible phosphatase PafA, originally identified in *Elizabethkingia meningoseptica* (23). PafA is a distinct PME that contains the pfam domain 01663, which is typically associated with PDEs but has greater activity toward phosphomonoesters (18). Indeed, PafA is most closely related to PhoD (Fig. 1*A*), an enzyme that is primarily a PDE (20, 36), while the Pi-irrepressible phosphatase identified in *Zymomonas mobilis*, originally labeled a PhoD (37), is also most closely related to *Bacteroidetes* PafA (Fig. 1*A*). We also scrutinized the genome of another model soil *Bacteroidetes* strain, *Chitinophaga pinensis,* identifying several homologs to the various PMEs (Fig. 1*A* and Table 1). It is noteworthy that *C. pinensis* encodes two distinct homologs of the PafA-type PME in its genome, both containing pfam01663.

**Table 1.** PMEs identified in *F. johnsoniae* DSM2064, *C. pinensis*, *P. putida* BIRD-1, and *E. coli*

Enzyme	Strain	Pfam domain	Gene name	Locus tag
PhoX	*F. johnsoniae*	ND*	*phoX^Fj^*	fjoh_2478
	*P. putida*	PF05787	*phoX^BIRD^*	PPUBIRD1_1093
PhoA	*F. johnsoniae*	PF00245	*phoA1^Fj^*	fjoh_3249
	*F. johnsoniae*	PF00245; PF13653	*phoA2^Fj^*	fjoh_3187
	*E. coli* (MG1655)	PF00245	*phoA^Ec^*	b0383
PafA	*F. johnsoniae*	PF01663	*pafA^Fj^*	fjoh_0023
	*C. pinensis*	PF01663	*pafA^Cp1^*	cpin_0724
	*C. pinensis*	PF01663	*pafA^Cp2^*	cpin_1665

*ND, not determined

To investigate the relative activity of these *Flavobacteria* PMEs and directly compare them with the classical PhoX (*Pseudomonas putida*) and PhoA (*Escherichia coli*) enzymes, we expressed the respective genes in a heterologous host (*P. putida* BIRD-1) mutated to lack its innate PME activity (Δ*phoX^BIRD^*::*Gm*) (38). The PME genes were under the control of the native *phoX* promoter and were expressed from the broad host range plasmid pBBR1MCS-km. The various complemented Δ*phoX^BIRD^*::*Gm* strains were grown overnight in a minimal medium containing a growth-limiting concentration (50 µM) of Pi (Fig. 1*B*). Given these enzymes are alkaline phosphatases, PME assays were performed using culture suspensions or by resuspending cells in a buffer (pH 9.4). We used the artificial substrate *para*-nitrophenyl phosphate (*p*NPP) as a proxy for PME activity, in which production of the colorimetric product *para*nitrophenyl (*p*NP) was quantified by spectroscopy. Plasmid copies of either *phoX*^BIRD^ (+pBX*phoX*^BIRD^) or the *E. coli phoA (*+pBX*phoA^Ec^*) restored PME activity to Δ*phoX^BIRD^*::*Gm* (Fig. 1*B*). Similarly, cells producing PhoA1^Fj^ (+pBX*phoA1^Fj^*), PhoA2^Fj^ (+pBX*phoA2^Fj^*), or PafA^Fj^ *(*+pBX*pafA^Fj^*) also restored PME activity, confirming their function as PMEs. Where PME activity was detected, all enzymes exhibited a preference for higher-pH conditions. Notably, when +pBX*pafA^Fj^* cells were resuspended in pH 9.4 buffer, significantly greater PME activity was detected in comparison to all other PMEs. Cells complemented with +pBX*phoX^Fj^* did not have any detectable PME activity, which could be due to incomplete expression of a fully folded and correctly localized lipoprotein. Another possibility for the lack of function in the *P. putida* heterologous host is that in all *Flavobacterium* genomes, the putative *phoX* is colocalized in an operon with a gene encoding a *c*-type cytochrome peroxidase similar to MauG (*fjoh_2477* in DSM2064). The role of MauG is to generate a protein-derived cofactor essential for the posttranslational modification and thus catalytic activity of methylamine dehydrogenase (39). Therefore, *Flavobacterium* PhoX may be unique in needing a cofactor to be functional. In support of this, *fjoh_2477* is highly expressed during Pi depletion along with *phoX^Fj^* (24).

The *phoX*^BIRD^ promoter was previously shown to be “leaky,” with low levels of PhoX^BIRD^ peptides detected in the exoproteome of cells grown on minimal medium under Pi-replete conditions. Consequently, when Δ*phoX^BIRD^*::*Gm* +pBX*phoA^Ec^*, +pBX*phoX*^BIRD^, +pBX*phoA1^Fj^*, and +pBX*phoA2^Fj^* were grown on Pi-replete solid minimal medium, detectable PME activity was observed, albeit lower than that under Pi-deplete conditions (*SI Appendix*, Fig. S1). Therefore, to investigate the potential Pi-insensitive function of PafA^Fj^, we compared the activity of all PMEs when grown in a complex medium. PME activity for +pBX*pafA^Fj^* was indeed significantly higher (up to 100-fold) than all other PMEs, confirming that PafA^Fj^ is a Pi-insensitive enzyme, while other PMEs, such as PhoX^BIRD^, are Pi sensitive (*SI Appendix*, Fig. S2).

### PafA Significantly Contributes toward PME Activity in *F. johnsoniae* DSM2064.

**Fig. 2.**
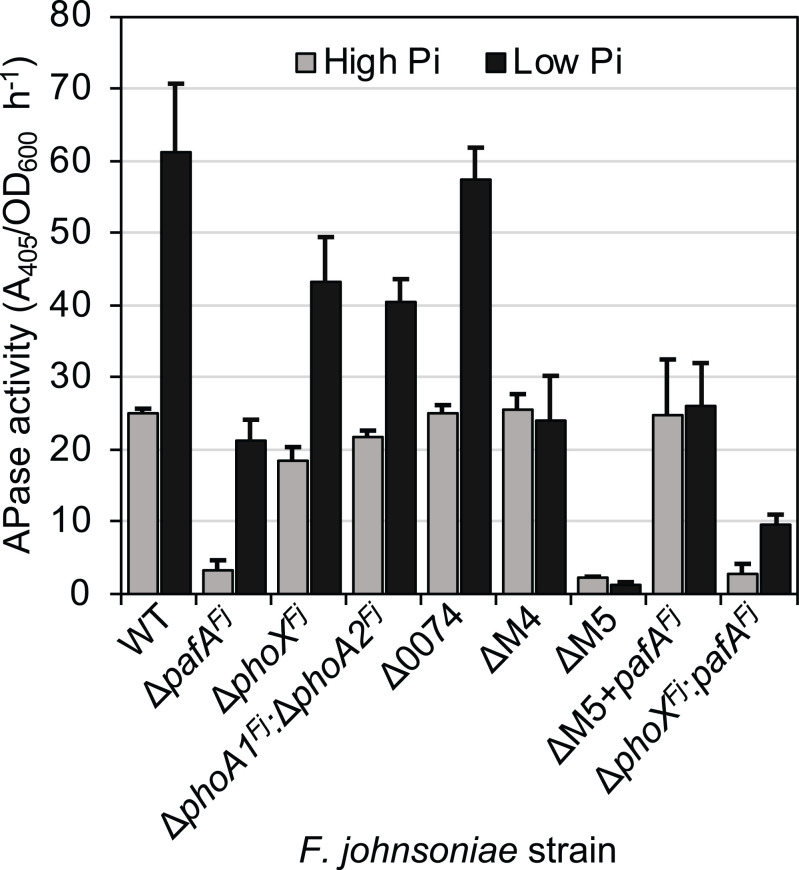
PME activity in wild-type and mutant strains of *F. johnsoniae*. PME activity was recorded in cell cultures (*n* = 3) grown overnight in minimal medium under phosphate-replete (High Pi = 1 mM) or phosphate-deplete (Low Pi = 50 μM) growth conditions. Values presented are the mean of biological triplicates, and error bars denote SD. Abbreviations: WT, wild type; Δ0074, Δ*fjoh_0074* single mutant; ΔM4, Δ*fjoh_0074:*Δ*phoX^Fj^*Δ*phoA1^Fj^:*Δ*phoA2^Fj^*; ΔM5, Δ*fjoh_0074:*Δ*phoX^Fj^:*Δ*phoA1^Fj^:*Δ*phoA2^Fj^:*Δ*pafA^Fj^*; ΔM5+pY:*pafA^Fj^*, ΔM5 complemented with *pafA^Fj^*.

Next, we examined the contribution of these PMEs toward activity in DSM2064, again using *p*NPP as an initial proxy. As expected (24), wild-type cells displayed both constitutive and elevated inducible PME activity in minimal medium under replete (1 mM) or deplete (50 µM) Pi levels, respectively (Fig. 2). Mutation of *pafA^Fj^* reduced PME activity under Pi-replete and Pi-deplete growth regimes, suggesting constitutive expression under both conditions. Mutation of Δ*phoX*^Fj^, encoding the distinct PhoX-like lipoprotein (Fig. 1*A*), reduced inducible PME activity in DSM2064. Likewise, a Δ*phoA1^Fj^:*Δ*phoA2^Fj^* double mutant also partially reduced inducible PME activity. We hypothesized that a gene (*fjoh_0074*) encoding a constitutively produced lipoprotein, annotated as a member of the endonuclease/exonuclease/phosphatase superfamily (InterPro#; PR005135), may make a minor contribution to PME activity under Pi-replete growth conditions. However, mutation of this gene had no observable effect on PME activity in DSM2064. To determine the contribution of PafA^Fj^ to both constitutive and inducible PME activity, we first generated a quadruple mutant, Δ*fjoh_0074:*Δ*phoX^Fj^:*Δ*phoA1^Fj^:*Δ*phoA2^Fj^* (ΔM4) and then a quintuple knockout mutant also lacking *pafA^Fj^* (ΔM5). PME activity measurements using these two mutant strains confirmed two important results. First, PafA^Fj^ is responsible for >95% of the constitutive, and approximately half of the inducible, PME activity. Second, the PMEs encoded by *phoX^Fj^*, *phoA1^Fj^*, and *phoA2^Fj^* are responsible for the additional inducible PME activity under Pi depletion. Complementation of the ΔM5 mutant with *pafA^Fj^* under the control of its native promoter (ΔM5 +pY:*pafA^Fj^*) duly restored PME activity to that comparable with the ΔM4 mutant in both conditions, confirming PafA^*Fj*^ is responsible for the majority of constitutive PME activity (Fig. 2). Finally, mutation of *pafA^Fj^* in the Δ*phoX^Fj^* background, creating a double mutant strain Δ*pafA^Fj^:*Δ*phoX^Fj^*, further confirmed a role for PhoX^Fj^ in inducible PME activity (Abs405/600 nm ⋅ h^−1^: Δ*pafA^Fj^* = 21± 2.8; Δ*pafA^Fj^:*Δ*phoX^Fj^* = 9.6 ± 1.2). Taken together, these results show that DSM2064 possesses four PMEs that contribute towards most of the PME activity in this bacterium, while the residual PME activity in the ΔM5 mutant indicates that at least one additional unidentified PME is encoded in its genome.

### PafA Enables Utilization of Organophosphorus Substrates as both a P and a C Source.

**Fig. 3.**
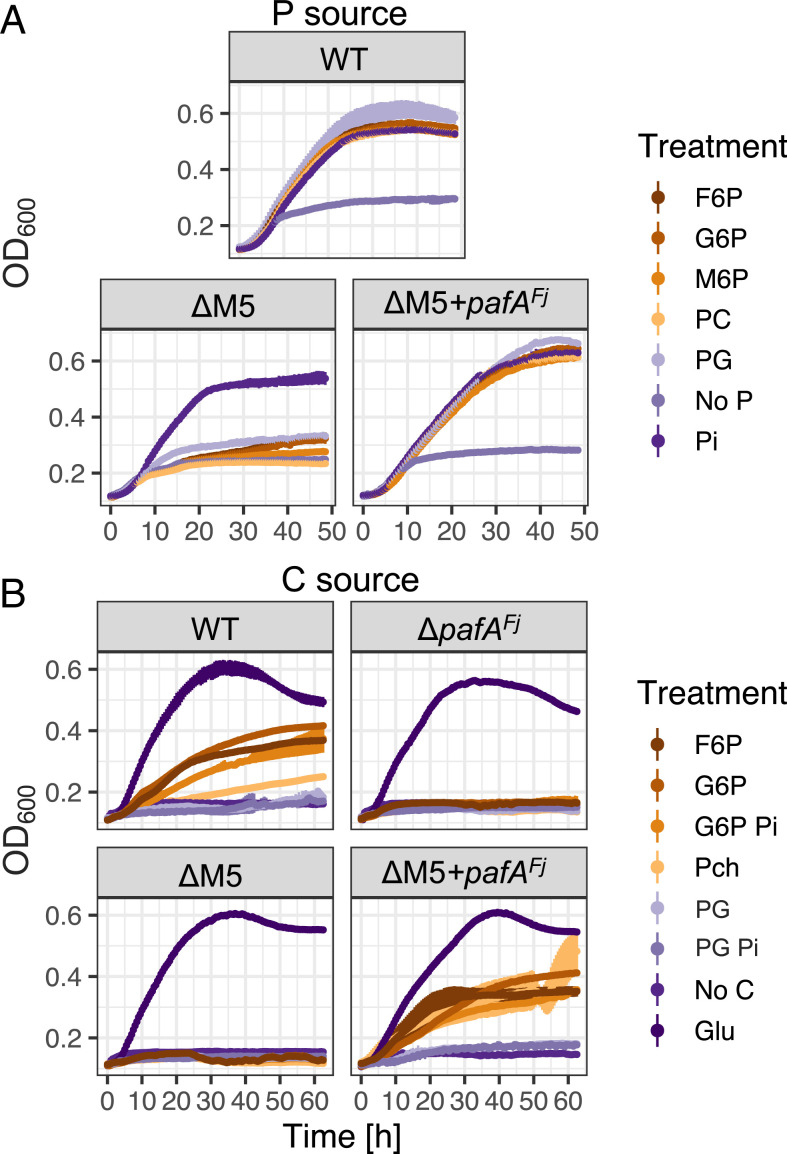
Growth of wild-type and mutant strains of *F. johnsoniae* DSM2064 on various organophosphorus substrates as a sole P and C source. (*A*) The wild type (WT), Δ*fjoh_0074:*Δ*phoX^Fj^:*Δ*phoA1^Fj^:*Δ*phoA2^Fj^*:Δ*pafA^Fj^* quintuple mutant (ΔM5), and *pafA-*complemented quintuple mutant (ΔM5+*pafA^Fj^*) were grown (*n* = 3) on various P substrates (200 µM) in addition to a no-P control. (*B*) The WT, Δ*pafA*, ΔM5, and ΔM5+*pafA^Fj^* were also grown (*n* = 3) on various P substrates (3 mM) as the sole C source in addition to no-C and glucose (Glu) controls. Results presented are the mean values, and error bars denote SD.

To determine the functional role of PafA^Fj^ in organophosphorus mineralization, we screened the parental wild type and selected PME mutant strains of DSM2064 (Fig. 3) on a range of organophosphorus substrates as the sole P source (200 μM) and glucose (10 mM) as the C source. Growth of wild-type DSM2064 on organophosphorus substrates was comparable to growth on Pi, while growth of the ΔM5 mutant was severely inhibited (Fig. 3*A*). In agreement with the very low level of PME activity in the ΔM5 mutant (Fig. 2), a small but detectable amount of growth did occur relative to the no-P control. The *pafA^Fj^*-complemented ΔM5 mutant fully restored the wild-type phenotype (Fig. 3*A*).

Next, we investigated whether PafA^Fj^ provides a novel mechanism for growth on organophosphorus substrates as a sole C, P, and energy source. To do this, organophosphorus substrates (3 mM) were individually supplied as the sole C and P source (Fig. 3*B*). A control condition with 15 mM glucose as the sole C source was also established. The wild type efficiently utilized phosphorylated carbohydrates (glucose 6-phosphate [G6P], mannose 6-phosphate [M6P], and fructose 6-phosphate [F6P]) and grew slowly on phosphocholine (PC) but could not utilize phosphoglycerol (PG). Given the latter two substrates were efficiently used as P sources (Fig. 3*A*), we attribute this low growth to a reduced or total inability to utilize the C moieties associated with PC and PG in contrast to phosphorylated carbohydrates. In addition, PG and G6P were provided as the sole C source in media supplemented with 1 mM Pi to inhibit expression/activity of inducible PMEs. However, this addition of exogenous Pi did not affect growth, suggesting PafA^Fj^ was the major enzyme responsible for catabolism of these compounds. Consistent with this, the Δ*pafA^Fj^* and ΔM5 mutants both failed to grow on these organic substrates as a sole C source, whereas the *pafA^Fj^*-complemented ΔM5 mutant grew like the wild type, confirming the essential role of PafA^Fj^ in the utilization of organophosphorus substrates as a sole C and energy source.

### Phosphate-Insensitive Organophosphorus Mineralization by PafA Liberates Bioavailable P.

**Fig. 4.**
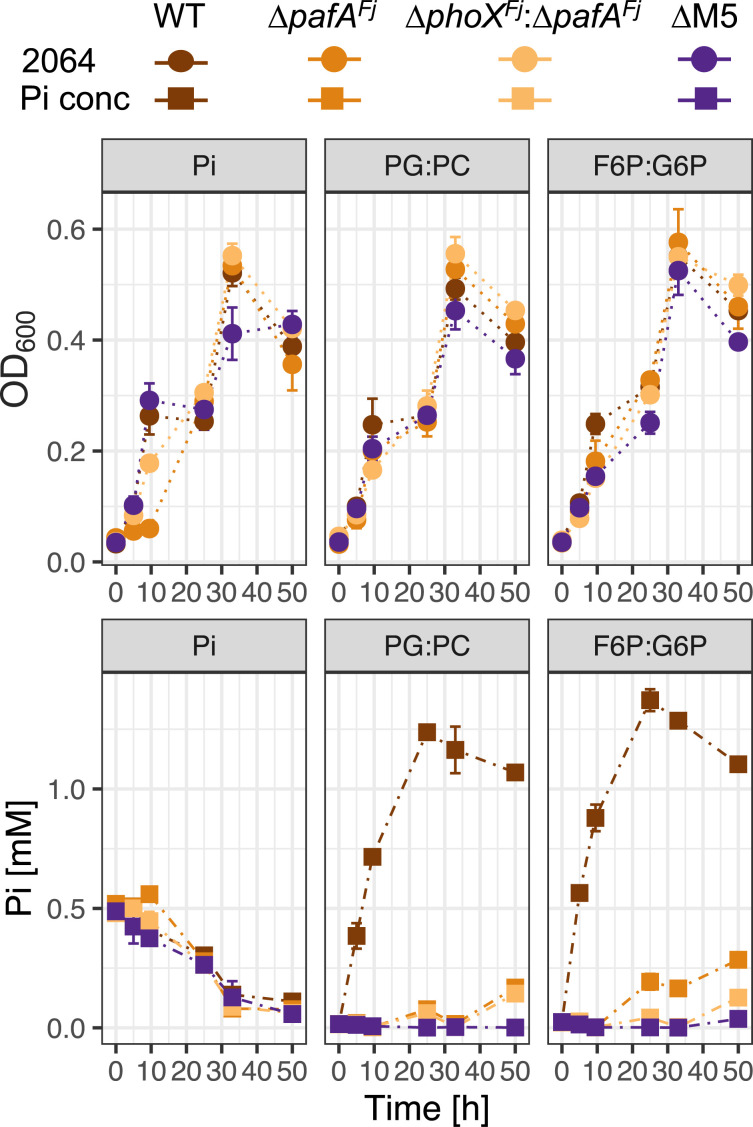
Organophosphorus mineralization and Pi export rates in wild-type and mutant strains of *F. johnsoniae* DSM2064. Wild type (WT) and the PME mutant strains, Δ*pafA^Fj^*, Δ*phoX^Fj^*:Δ*pafA^Fj^*, and Δ*fjoh_0074:*Δ*phoX^Fj^:*Δ*phoA1^Fj^:*Δ*phoA2^Fj^*:Δ*pafA^Fj^* (ΔM5) were grown in the presence of glucose (5 mM) and an organophosphorus substrate mix (2 mM). After 25 h, an additional 5 mM glucose was added to all cultures. Growth (2064, circles, *Upper*) and Pi accumulation (Pi conc, squares, *Lower*) were quantifiedover time. Data points represents the mean of duplicate cultures sampled in duplicate, and error bars denote the SD.

To investigate whether phosphate-insensitive mineralization of organophosphorus contributes toward the regeneration of bioavailable Pi, the concentration of exogenous Pi in culture supernatants was quantified during growth on various phosphomonoesters. DSM2064 wild type and the various PME mutants (Δ*pafA^Fj^*, Δ*pafA^Fj^:*Δ*phoX^Fj^*, or ΔM5) were individually grown in minimal medium containing 5 mM glucose supplemented with either 2 mM PG and PC (50:50 mix) or 2 mM phosphorylated carbohydrates (50:50 mix G6P:F6P), as well as a Pi control (500 μM). After 25 h, cultures were supplemented with additional glucose (estimated final concentration: 5 mM). Wild-type DSM2064 cultures rapidly remineralized each organophosphorus mix, resulting in the accumulation of exogenous Pi (Fig. 4, *Lower*). In contrast, the ΔM5 mutant cultures showed no sign of Pi accumulation. We attribute the growth of the ΔM5 mutant in both organophosphorus treatments (Fig. 4, *Upper*) to a small but detectable concentration of Pi contamination (14 to 24 μM, Fig.4) at T0 that was exhausted during incubation. The cultures of the Δ*pafA^Fj^* and Δ*pafA^Fj^*:Δ*phoX^Fj^* mutants accumulated small quantities of Pi, suggesting that low levels of mineralization by phosphate-sensitive PMEs (PhoX, PhoA1, and PhoA2) occurred.

We confirmed that liberation of bioavailable Pi from organophosphorus could support growth of another bacterium by taking spent growth medium used to grow wild-type DSM2064, filtering out the cells, and diluting 50:50 with fresh medium lacking a P source. Two conditioned mediums were produced, one using spent medium from cells grown on 5 mM glucose and 2 mM G6P (C:*P* = 21:1) and another using spent medium from cells grown on 7 mM glucose and 50 µM Pi (C:P = 840:1). The *P. putida* Δ*phoX^BIRD^*::*Gm* and DSM2064 ΔM5 mutants, both incapable of organophosphorus mineralization, were then individually inoculated into each conditioned medium. A no-bacterial-inoculant culture and a wild-type DSM2064-inoculated culture were established as negative and positive controls, respectively. All three strains grew when conditioned medium was generated from G6P-grown cultures but not when conditioned medium was generated from Pi-grown cultures (*SI Appendix*, Fig. S3 for full details). Together, these data reveal that Pi-insensitive mineralization of organophosphorus by PafA^Fj^ results in the rapid accumulation of labile Pi that can support secondary growth of other bacteria.

### PafA Is Predominantly Affiliated with *Bacteroidetes*.

**Fig. 5.**
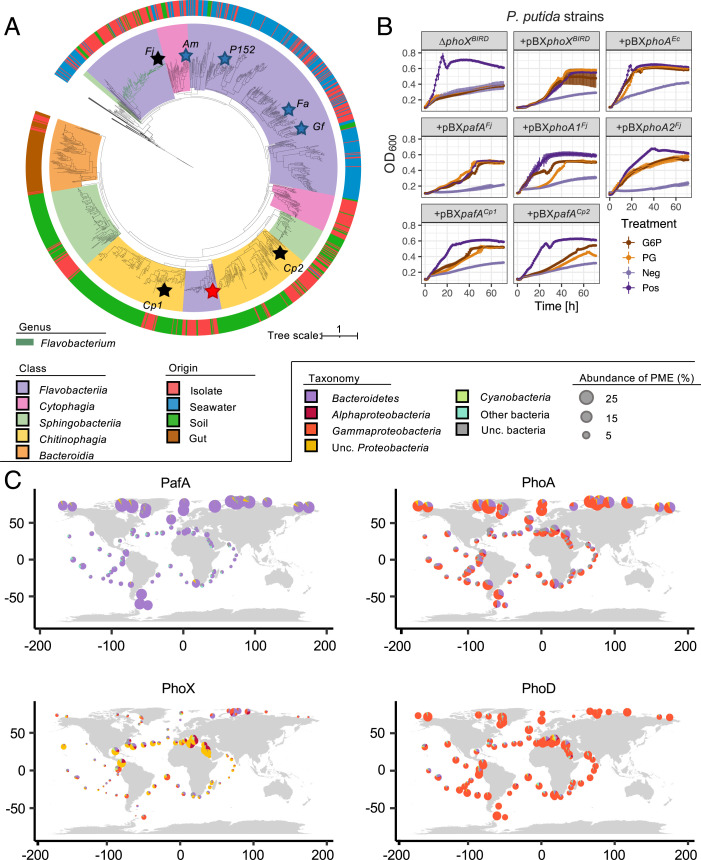
Environmental diversity of PafA and other alkaline PMEs in soil, gut, and ocean microbiomes. (*A*) PafA diversity in genome-sequenced isolates and environmental metagenomes (outer ring denotes origin). PafA sequences related to each class of *Bacteroidetes* are colored. Branches corresponding to the genus *Flavobacterium* are highlighted in green. Black stars represent the two *C. pinensis* PafA homologs heterologously produced in *P. putida*. The red star denotes the characterized PafA found in *E. meningoseptica*. The blue stars denote marine Flavobacteria strains, namely *A. machipongonensis* PR1 (Am), *F. agariphila* KMM 3901 (Fa), *Polaribacter* sp. MED152 (P152), and *G. forsetii* KT0803 (Gf), assayed for PME activity during growth in complex medium (marine broth) (Table 2). (*B*) Growth of the *P. putida* Δ*phoX^BIRD^* mutant complemented with +pBX*phoX^BIRD^*, +pBX*phoA^Ec^*, +pBX*phoA1^Fj^*, +pBX*phoA2^Fj^*, +pBX*pafA^Fj^*, +*pBXpafA^Cp1^*, or +*pBXpafA^Cp2^* on organophosphorus compounds (200 μM) as the sole P source. Results presented are the mean of triplicate cultures. Error bars denote SD. (*C*) Distribution of the four major alkaline PMEs in the global ocean (surface water only) based on the Tara Oceans dataset. The area of each pie chart represents the normalized gene abundance at each sampling site (Dataset S2) expressed as the percentage of bacteria possessing a given PME (see legend for scaling) and the contributing taxa.

While the majority of inducible PME encoding genes are predominantly found in the genomes of plant-associated *Bacteroidetes*, the occurrence of *pafA* homologs in bacteria related to this phylum is more widespread (24). To further investigate the environmental distribution of *pafA* open reading frames (ORFs), we scrutinized various marine (Tara Oceans), plant rhizosphere (oilseed rape and wheat), and gut (rumen and human) metagenomes deposited in the Integrated Microbial Genomes & Microbiomes server at the Joint Genome Institute (IMG/JGI) database (total *n* = 357). We combined the retrieved environmental sequences with *pafA* ORFs previously identified in our *Bacteroidetes* isolate genome bank (*n* = 468) (24). Only ORFs encoding enzymes possessing key amino acid residues required for PME activity (18, 24) were retained (environmental *n* = 1,314; isolate *n* = 423) for phylogenetic comparison (Fig. 5*A* and Dataset S1). In addition to the *Bacteroidetes* PafA hits, we also identified environmental homologs (*n* = 233) that fell outside the *Bacteroidetes* group. Using the Reference Sequence (RefSeq) database (BLASTp), we identified that these sequences belonged to *Acidobacteria*, *Candidatus* Lindowbacteria, V*errucomicrobia*, *Gemmatimonadetes*, and *Planctomycetes* (*SI Appendix*, Fig. S5 and Dataset S1). Phylogenetic analysis revealed the existence of several polyphyletic subclades, as evidenced by the occurrence of two distinct PafA-encoding ORFs (Cpin_0724 and Cpin_1665) in the genome of *C. pinensis* (Cp1 and Cp2 in Fig. 5*A*). These subclades appeared to be largely separated by their environmental origin (marine, gut, and soil), except for terrestrial *Flavobacterium* PafA ORFs. To confirm the function of the PafA homologs within these branches, we cloned the *C. pinensis* genes encoding Cp1 and Cp2 and introduced the resulting plasmids into Δ*phoX^BIRD^::Gm,* demonstrating both are functional Pi-insensitive PMEs (*SI Appendix*, Fig. S4) that can mineralize naturally occurring organophosphorus molecules (Fig. 5*B*).

**Table 2.** PME activity of marine bacteria grown overnight (*n* = 3) in marine broth (complex medium, phosphate replete), obtained through addition of the artificial substrate *p*NPP (10 mM)

Strain	Phylum	Class	PME activity (h^−1^)
*Roseobacter denitrificans* OCh 114	*Proteobacteria*	*Alphaproteobacteria*	0.033 ± 0.044
*Ruegeria pomeroyi* DSS-3	*Proteobacteria*	*Alphaproteobacteria*	0.001 ± 0.019
*Dinoroseobacter shibae* DFL-12	*Proteobacteria*	*Alphaproteobacteria*	0.055 ± 0.057
*Algoriphagus machipongonensis* PR1^†^	*Bacteroidetes*	*Cytophagia*	3.214 ± 0.069
*Formosa agariphila* KMM 3901^†^	*Bacteroidetes*	*Flavobacteriia*	2.335 ± 0.089
*Polaribacter* sp. MED152^†^	*Bacteroidetes*	*Flavobacteriia*	1.510 ± 0.043
*Gramella forsetii* KT0803^†^	*Bacteroidetes*	*Flavobacteriia*	0.509 ± 0.070*

*Poor growth and cell aggregation in culture medium.

^†^Possession of *pafA* in the genome.

Sequences related to soil-/plant-associated *Flavobacterium* PafA represented a small fraction of the overall PafA diversity, and few environmental sequences were retrieved from the soil/rhizosphere metagenomes associated with the two crop species. A large proportion of diversity belonged to marine *Flavobacteriia* (predominantly the family *Flavobacteriaceae*), including numerous sequences captured from the Tara Oceans dataset. Screening four marine *Bacteroidetes* and three marine *Alphaproteobacteria* showed that possession of *pafA* correlated with Pi-insensitive PME activity (i.e., all four marine PafA-encoding *Bacteroidetes* showed high PME activity even in high P medium), whereas all three non PafA-encoding *Alphaproteobacteria* displayed PME activity that was inhibited in Pi-rich medium (Table 1). All gut-related PafA sequences clustered closely with isolates related to *Bacteroidales*, including *Prevotella* spp. The previously characterized PafA from *E. meningoseptica* was in a cluster associated with *Chryseobacterium* sequences more closely related to *Sphingobacteraceae* homologs than those from *Flavobacteraceae* (Fig. 5*A*). These data suggest PafA may have undergone some level of environmental adaptation after its first appearance.

### PafA and PhoA Are Highly Prevalent in the Global Ocean.

We next compared the abundance, expression, and associated phylogeny of the genes encoding the three major classes of bacterial alkaline PMEs (PhoA, PhoD, and PhoX) and PafA in the global ocean. Unexpectedly, both *pafA* and *phoA* were more prevalent than *phoX* in surface samples across numerous oceanic sites, particularly those in polar and temperate regions of the ocean (Fig. 5*C* and Dataset S2). While comparison of transcription levels between genes does not necessarily equate to functional enzyme activity, nor account for differences in corresponding gene abundance, we also discovered that *pafA* and *phoA* were more highly transcribed in surface samples within these regions (*SI Appendix*, Fig. S6 and Dataset S2). For example, in the epipelagic (surface water and deep chlorophyll maximum) across the Arctic Ocean and Southern Ocean, *pafA* and *phoA* transcription was over 10-fold greater than *phoX* (*SI Appendix*, Fig. S7). In the mesopelagic, *pafA* was present in 5 to 10% of bacterial cells, which was comparable to or greater than *phoX* at all sites (*SI Appendix*, Fig. S7). However, across the Pacific Ocean and South Atlantic Ocean combined, *phoA*, *phoX*, and *phoD* all had significantly higher (*SI Appendix*, Table S3) transcript abundances than *pafA* despite similar gene abundance profiles at these sites. As expected, most *pafA* sequences were related to *Bacteroidetes.* We also found an unexpected abundance of *phoD* sequences across most oceanic sites, the majority of which were related to marine *Gammaproteobacteria*. Given these unexpected results that contradict previous work (14), we also analyzed the Tara Oceans database using the latter bioinformatics pipeline described by Sebastian et al. (14). This revealed that BLASTp analysis dramatically underestimated the prevalence of *phoA* (*SI Appendix*, Table S4) by failing to capture the full diversity of *phoA* sequences present in the global ocean (*SI Appendix*, Figs. S8 and S9). Transcript abundance for genes encoding the canonical PMEs, PhoX, PhoA, and PhoD was highest in the regions typified by very low concentrations of Pi (40) (i.e., the Mediterranean Sea [MS] [*SI Appendix*, Fig. S6]. Regression analysis combining all sampling sites (with recorded Pi concentrations) and depths (epi- and mesopelagic) confirmed global *phoX* prevalence (*R*^2^ = 0.1804, *P* < 0.001), and transcription (*R*^2^ = 0.05137, *P* < 0.01) was negatively correlated with standing stock concentrations of Pi (*SI Appendix*, Fig. S10). On the other hand, global *pafA* prevalence (*R*^2^ = 0.1296, *P* < 0.001) and transcription (*R*^2^ = 0.05432, *P* < 0.01) were positively correlated with Pi, demonstrating the Pi-independent functional role of this enzyme. In contrast, *phoD* gene abundance and expression were both negatively correlated. The prevalence of *phoA* did not correlate with Pi (*R*^2^ = 0.0028, *P* = 0.24), though transcription was positively correlated with Pi (*R*^2^ = 0.06746, *P* < 0.001). In summary, *pafA* and *Flavobacteriia*-like *phoA* sequences are diverse and widespread in nature, including in soil, gut, and ocean microbiomes, with PafA representing a major enzyme in the global P cycle.

## Discussion

We report that a unique class of PME, termed PafA, is abundant in nature and enables the rapid mineralization of various organophosphorus substrates independently of exogenous Pi concentration. Despite *Flavobacterium* spp. possessing redundancy for PMEs, encoding PafA, PhoX, and two PhoA type enzymes, PafA was essential for growth on phosphorylated carbohydrates as a sole C or sole C and P source, revealing functional diversification of PafA compared to other well-known phosphatases (19–22, 41). These findings are consistent with the Pi-insensitive expression of *pafA* and enzyme activity that is not inhibited by Pi (18, 23, 24). Despite relatively low expression of *pafA* in *Flavobacterium* spp. during Pi-replete or Pi-deplete growth conditions (24), we show that this enzyme is responsible for the rapid conversion of various natural organophosphorus substrates into bioavailable Pi independently of P availability. Thus, we reveal another widespread Pi-insensitive mechanism for the rapid conversion of organophosphorus into bioavailable P (42, 43), which may explain why PME activity is detected in Pi-replete oceanic regions (4, 44, 45). Significantly, unlike genes encoding the canonical PMEs, *phoX*, *phoD*, and *phoA*, *pafA* distribution and transcription in seawater are almost exclusively affiliated with *Bacteroidetes*, which frequently associate with phytoplankton and sinking particles (46, 47), suggesting that this phylum plays an overlooked and potentially major role in remineralizing labile Pi.

PhoX is considered the most abundant phosphatase in the ocean (14), with iron limitation affecting this enzyme’s efficacy and limiting microbial Pi mineralization (48). However, genes encoding distinct PhoA homologs that do not require iron for catalytic activity (18) are also prevalent in the global ocean (17). Our comparative analysis of the four major bacterial alkaline phosphatases in the global ocean revealed that *Gammaproteobacteria* and *Bacteroidetes*, through expression of *pafA*, *phoA*, and *phoD*, are key players in the remineralization of organophosphorus, dwarfing the role of *phoX*-harboring *Alphaproteobacteria* across most oceanic sampling sites but particularly in polar regions. This suggests a greater role for organophosphorus remineralization in marine P cycling via a mechanism that is not as constrained by Pi standing stock concentrations as was previously thought (14, 21, 40). While extrapolation from our single-organism laboratory-based experiments could be misleading, the difference between *phoX* and *pafA* expression profiles across the global ocean further suggests that PafA has a functional role greater than that of scavenging P [i.e., a role in C-utilization (19, 21)]. In addition, transcriptional profiles for *phoA* also suggest that the function of this enzyme may have diversified, unlike *phoX*. In agreement, the transcriptomic and proteomic data in our previous study (24) and experimental data presented here show no essential role for PhoA1^Fj^ or PhoA2^Fj^ in P scavenging from phosphomonoesters. Given PafA and PhoA can also mineralize phosphodiesters and phosphotriesters (17, 18), these enzymes may play a large and previously unrecognized role in environmental P cycling. PafA homologs were also found in the genomes of abundant oligotrophic soil and marine bacteria related to *Acidobacteria*, *Candidatus* Lindowbacteria, V*errucomicrobia, Gemmatimonadetes*, and *Planctomycetes*; future research is required to ascertain their activity and sensitivity toward Pi to improve our understanding of global P cycling.

*Bacteroidetes* are major organic polymer degraders that are typically associated with algal, plant, and animal related niches, with their success in diverse environments driven by their ability to coexist through divergent C-acquisition strategies (26, 49). Our data reveals another unique strategy for scavenging organic C from various phosphorylated molecules that are abundant in nature (5, 50). In ocean and plant microbiomes, this metabolism may provide a competitive advantage for C acquisition when residual Pi levels inhibit PME activity, rendering these molecules inaccessible to bacteria that lack PafA. In animals, nutritional utilization of phosphorylated carbohydrates and other organophosphorus substrates, as well as simple sugars, plays a significant role in various pathogen–host interactions, with uptake of these nutrients typically requiring the presence of specialized transporters (51–54). This often precludes the requirement for extracellular phosphatases that are not synthesized or whose activity is inhibited by exogenous Pi (14, 19, 20, 22, 23). *Bacteroidetes* are unique among bacteria, lacking most ABC transporters required for the uptake of organic molecules. Therefore, we speculate that PafA may have evolved early in this lineage to compensate for this lack of ABC transport systems, which would explain both the ubiquitous occurrence of this enzyme across this phylum and why it is not associated with a particular environmental niche, unlike other *Bacteroidetes* PMEs (24). Importantly, preferential use of phosphorylated carbohydrates as C and energy sources and subsequent release of mineralized Pi may drive P flux in systems dominated by *Bacteroidetes* such that their role in environmental P cycling may be comparable to their role in C cycling (4, 26, 31).

PMEs are commercially utilized in agriculture to improve the nutritional value of animal grain feed, and interest in their application to release bioavailable Pi in soils is growing (55, 56). A major limitation of PMEs is their limited substrate specificity and/or their inhibition by exogenous Pi. Our demonstration that *Flavobacterium* PafA possesses high activity toward both artificial and natural organophosphorus substrates and is easily produced in a heterologous host highlights the promise of this enzyme for biotechnological applications such as improving the nutritional value of animal and plant feed, developing sustainable agriculture, and reducing our reliance on unsustainable chemical P fertilizers (1, 5). Given other plant-associated *Flavobacterium* spp. display comparable or greater PME activity (24), the PafA enzymes from these strains may be even better candidates for commercial use; further investigation should ascertain the structure–function relationships responsible for the apparent differences in PME activity between PafA homologs.

In summary, this study resolved the contribution of seemingly redundant PMEs toward growth on organophosphorus substrates as sole C, P, and energy sources in plant-associated *Flavobacterium* spp. The emergence of PafA as a highly active, Pi-insensitive PME facilitating the rapid mineralization of bioavailable Pi that is widespread in nature uncovers a major player in the global P cycle.

## Materials and Methods

### Growth and Maintenance of Bacterial Strains.

*F. johnsoniae* DSM2064 (UW101) was purchased from the Deutsche Sammlung von Mikroorganismen und Zellkulturen (DSMZ) collection. The *P. putida* BIRD-1 *phoX* knockout mutant was generated previously (38). The marine *Bacteroidetes* spp., *Algoriphagus machipongonensis* PR1 (DSM24695), *Formosa agariphila* KMM 3901 (DSM15362), and Roseobacter strains were also obtained from the DSMZ collection, whereas *Polaribacter* sp. MED152 and *Gramella forsetii* KT0803 were kindly obtained from Jarone Pinhassi, Linnaeus University, Kalmar, Sweden, and Jörg Wulf, Max Planck Institute for Marine Microbiology, Bremen, Germany, respectively.

*F. johnsoniae* and *P. putida* strains were routinely maintained on casitone yeast extract medium (CYE) (52) containing casitone (4 g ⋅ L^−1^), yeast extract (1.25 g ⋅ L^−1^), MgCl_2_ (350 mg ⋅ L^−1^), and agar (20 g ⋅ L^−1^) or lysogeny broth (LB) containing agar (15 g ⋅ L^−1^), respectively. For various growth experiments and phosphatase assays, *F. johnsoniae* was grown in a minimal A medium adapted from ref. 19. This medium contained glucose (5 to 20 mM), NaCl (200 mg ⋅ L^−1^), NH_4_Cl (450 mg ⋅ L^−1^), CaCl_2_ (200 mg ⋅ L^−1^), KCl (300 mg ⋅ L^−1^), MgCl_2_ (450 mg ⋅ L^−1^), FeCl_2_ (10 mg ⋅ L^−1^), MnCl_2_ (10 mg ⋅ L^−1^), 20 mM Bis/Tris buffer pH 7.2, and KH_2_PO_4_ added to a final concentration ranging from 50 μM to 1 mM. For *P. putida* strains, glucose was replaced with sodium succinate (final concentration: 15 mM). Marine *Bacteroidetes* and Roseobacter strains were grown in Difco Marine Broth 2216 (Fisher Scientific) and incubated at 28 °C.

The organophosphorus substrates F6P (Chemical Abstracts Service [CAS]: 103213-47-4), G6P (CAS: 3671-99-6), M6P (CAS: 33068-18-7), PG consisting of a mix of glycerol 2-phosphate (CAS: 55073-41-1) and glycerol 3-phosphate (CAS: 29849-82-9), PC (CAS: 72556-74-2), and L-α-phosphatidylinositol (CAS: 97281-52-2) were purchased from Sigma-Aldrich, Merck. A final concentration of 200 to 500 μM was used for growth experiments with organophosphorus substrates as a sole P source. For growth experiments using organophosphorus substrates as a sole C source or sole C and P source, a final concentration of 2 to 3 mM was used.

For generating conditioned medium, glucose (5 mM) and either 1 mM G6P or 100 μM Pi was used. After overnight growth, supernatants were collected after removing cells by centrifugation (10,000 × *g*) and filtration through a polyethersulfone membrane (0.22-μm pore size). Spent medium was mixed with the same volume of fresh medium containing 5 mM glucose, and half of the subsequent culture lines were supplemented with 250 μM Pi (positive control).

### Construction of *Flavobacterium* Mutants and Subsequent Complementation.

To construct the various PME mutants in DSM2064, the method developed in ref. 57 was used. A full list of primers used in this study can be found in *SI Appendix*, Table S1. Briefly, two 1- to 1.6-kb regions flanking each gene were cloned into plasmid pYT313 using the HiFi assembly kit (New England Biosciences). Sequence integrity was checked via sequencing. The resulting plasmids were transformed into the donor strain *E. coli* S17-1 λ*pir* (S17-1 λ*pir*) and mobilized into *Flavobacterium* via conjugation. Briefly, 1 mL overnight culture was inoculated into fresh 5 mL CYE or LB media and incubated for 8 h. Cells were individually washed in 1 mL CYE, and a 200 μL donor:recipient (CYE) suspension (1:1) was spotted onto CYE containing CaCl_2_ (0.6 g ⋅ L^−1^) and incubated overnight at 30 °C. Biofilm was scraped from the agar surface and resuspended in 1 mL minimal A medium (no C source). Transconjugants were selected by spreading 5 to 100 μL aliquots on CYE containing erythromycin (100 μg ⋅ mL^−1^). Colonies were restreaked onto CYE erythromycin plates to remove any background wild type. Single homologous recombination events were confirmed by PCR prior to overnight growth in CYE followed by plating onto CYE containing 10% (wt/vol) sucrose to select for a second recombination event resulting in plasmid excision. To identify a double homologous recombination, mutant colonies were replica plated onto CYE containing 10% (wt/vol) sucrose and CYE containing erythromycin (100 μg ⋅ mL^−1^). Erythromycin-sensitive colonies were screened by PCR.

For complementation of the ΔM5 mutant, the replicative plasmid pCP11 was used. Briefly, *pafA* (fjoh_0023) and its 350-bp upstream region were cloned into pCP11 using the HiFi assembly kit. Proper plasmid construction was confirmed by Sanger sequencing. The Plasmid pCP:*pafA^Fj^* was mobilized into DSM2064 via conjugation using S17-1 λ*pir* as the donor strain. The method was identical to that described for transfer of the suicide plasmid, pYT313, except that 1 mL overnight cultures of donor and recipient were directly washed and resuspended in 200 μL CYE prior to spotting onto CYE containing CaCl_2_ (0.6 g ⋅ L^−1^). Cells were scraped from the solid medium and transformants selected by creating a serial dilution (10^−1^ to 10^−5^) from the cell suspension and spotting 20 μL of each dilution onto CYE containing erythromycin (100 μg ⋅ mL^−1^).

### Construction of *P. putida* BIRD-1 Strains.

To complement the *Pseudomonas* sp. BIRD1 Δ*phoX* mutant with *Bacteroidetes* phosphatases, the promoter for the native *phoX*:BIRD-1 was cloned into the broad-host range plasmid pBBR1MCS-km using methods outlined in ref. 38. The *Flavobacterium* PMEs were subsequently cloned downstream of this promoter using the HiFi assembly kit (New England Biosciences). The genes encoding PafA homologs from *C. pinensis* DSM2588 (CP1, IMG gene accession 644962876; CP2, IMG gene accession 644963845) were synthesized (Integrated DNA Technologies) with HindIII and XbaI restriction sites added at the 5′ and 3′ ends, respectively. After digestion of the fragment and plasmid, ligation was performed using T4 DNA ligase (Promega). Plasmids were mobilized into the Δ*phoX* mutant via electroporation using a voltage of 18 kV ⋅ cm^−1^ or by biparental mating with the *E*. *coli* donor strain S17-1 λ*pir.* For electroporation, cells were immediately added to LB and incubated for 2 to 3 h prior to selection on LB supplemented with 50 μg ⋅ mL^−1^ kanamycin. For conjugative plasmid transfer, S17-1 λ*pir* and *P. putida* (Δ*phoX*) were grown overnight in LB broth (0.5 mL) and resuspended in 0.1 mL fresh medium. Strains were mixed and spotted onto LB agar and incubated for 5 h. Cells were scraped from solid medium and resuspended in 1 mL Tris HCl buffer (pH 7.4). A 1:10 serial dilution was established and plated onto LB supplemented with 50 μg ⋅ mL^−1^ kanamycin and 10 μg ⋅ mL^−1^ gentamicin to counterselect against S17-1 λ*pir*. Colonies were screened by PCR for the presence of the plasmid.

### Quantification of Alkaline Phosphatase Activity.

The protocol was adapted from ref. 24, with volumes adjusted for compatibility with a microtiter plate reader (Tecan SPARK 10M). Cell cultures (*n* = 3) for both Pi-replete and Pi-deplete growth conditions were directly incubated (30 °C at 160 rpm) with 10 mM (final concentration) *p*NPP or resuspended in a Tris⋅HCl buffer adjusted to pH 5.4, 7.4, or 9.4 prior to *p*NPP incubations. All reactions were incubated at 28 °C on a rotary shaker (230 rpm). The reaction was stopped using 2 mM (final concentration) NaOH once visible production of the colorimetric product *p*NP was observed, typically when 10 to 20% of the reaction had occurred. For each strain and growth condition, absorbance at 405 nm (A_405_) measurements were corrected by subtracting A_405_ measurements for reactions immediately stopped with NaOH. Normalization against the culture optical density at 600 nm was performed, and the rate was calculated and expressed in units per hour (h^−1^).

### Quantification of Exogenous Phosphate.

To quantify Pi mineralization in the presence of organic P, cells were grown in a minimal medium supplemented with glucose (5 mM) and 1 mM each of PG and PC or 1 mM each of F6P and G6P. A control using 500 μM Pi was also performed. To quantify exogenous Pi, cells were removed from culture aliquots via centrifugation (10000 × *g* for 5 min), and Pi concentrations in the supernatants were determined according to the method of Chen et al. (58) modified for compatibility with a microtiter plate reader. Briefly, 100 μL supernatant was added to 100 μL dH_2_O:6N sulfuric acid:2.5% (wt/vol) ammonium molybdate:10% (wt/vol) ascorbic acid in a 2:1:1:1 ratio. For each individual assay, absolute quantification of Pi was achieved using a standard curve of known Pi concentrations (0, 7.81, 15.63, 31.25, 62.50, 125, 250, and 500 μM). This enabled a reduced incubation time at 37 °C of 30 to 45 min. Absorbance was measured at 820 nm. Each *F. johnsoniae* strain, wild type plus three PME mutants, were grown under the different treatments in duplicate cultures with duplicate technical replicates for growth and Pi concentrations taken for each culture (*n* = 4 total).

### Bioinformatics Analyses.

The online platform IMG/JGI was used to perform most comparative genomics analyses described in this study. Genomes and metagenomes were stored in genome sets, and for PafA, BLASTp searches (minimum similarity 30%, E-value e^−50^) were set up using the “jobs function.” The diversity, richness, and gene and transcript abundance of *phoA*, *phoD*, *phoX*, and *pafA* in seawater was determined by searching the Tara ocean metagenome (OM-RGC_v2_metaG) and metatranscriptome (OM-RGC_v2_metaT) databases via the Ocean Gene Atlas web interface, using the hmmsearch function (stringency 1E^−60^). Profile Hidden Markov Models (pHMM) for PhoA (PF00245), PhoX (PF05787), and PhoD (PF PF09423) ORFs were downloaded from https://pfam.xfam.org/. For PafA, a pHMM was manually curated by aligning sequences using multiple sequence comparison by log-expectation (MUSCLE) identified in various *Bacteroidetes* isolates and pHMM using the hmmbuild function in hmmer 3.3 (hmmer.org). Sequence abundances were expressed as the average percentage of genomes containing a gene copy or transcript by dividing the percentage of total mapped reads by the median abundance (as a percentage of total mapped reads) of 10 single-copy marker genes (59) for both metagenomic and metatranscriptomic datasets.

To determine the phylogeny of PafA, sequences were aligned using MUSCLE and manually inspected for the possession of key amino acid residues using Molecular Evolutionary Genetics Analysis software version X (MEGAX). Sequences possessing any point mutations at sites of key residues were removed from the multiple alignment. Phylogenetic reconstruction was performed using IQ-Tree using the parameters -m TEST -bb 1000 -alrt 1000. Evolutionary distances were inferred using maximum-likelihood analysis. Relationships were visualized using the online platform the Interactive Tree of Life viewer (https://itol.embl.de/).

All statistical analyses and data visualizations were performed using the ggplot2, ggfortify, tidyr, plyr, serration, and rcolorbrewer packages in Rstudio (1.2.5033).

## Supplementary Material

Supplementary File

Supplementary File

Supplementary File

## Data Availability

The oilseed rape metagenomes generated in this study have been deposited in Genbank under the accession no. PRJNA738866. All other publically available metaomics datasets were accessed via the IMG/JGI (https://img.jgi.doe.gov/) and Ocean Gene Atlas (https://tara-oceans.mio.osupytheas.fr/) online portals.
