# Supplementary material for: A widely distributed phosphate-insensitive phosphatase presents a route for rapid organophosphorus remineralization in the biosphere
Source: Proc Natl Acad Sci U S A. 2022 Jan 26;119(5):e2118122119. doi: 10.1073/pnas.2118122119 (PMC8812569; doi:10.1073/pnas.2118122119)
Supplement: Supplementary File [file pnas.2118122119.sapp.pdf]

A widely distributed phosphate-insensitive phosphatase presents a route for rapid organophosphorus remineralisation in the biosphere

Ian D.E.A. Lidbury[1*], David J. Scanlan[2], Andrew R. J. Murphy[2], Joseph A. Christie-Oleza[3], Maria M. Aguilo-Ferretjans[3], Andrew Hitchcock[1], Tim Daniell[1]

[1]Plants, Photosynthesis, and soil, School of Biosciences, University of Sheffield, Sheffield, UK

[2]School of Life Sciences, University of Warwick, Gibbet Hill Road, Coventry, UK

[3]University of the Balearic Islands, Palma, Spain

Corresponding author: **I.lidbury@sheffield.ac.uk**

Supplementary Tables

Table S1. List of primers used for mutagenesis of *Flavobacterium johnsoniae* DSM2064

Primer	Sequence 5'-3'	Function
KO2064_3187_AF	GCAGCGGAAAAATTCGGGGGATCCTTGCATTCAGGTTTTCATTAGCG	For primer region A – PhoA2
KO2064_3187_AR	TTCCGGGACCAAAGTTCCAGTGCTCATTCCGTC	Rev primer region A – PhoA2
KO2064_3187_BF	TGAGCACTGGAACTTTGGTCCCGGAAGTGAAC	For primer region B – PhoA2
KO2064_3187_BR	ATTACGCCAAGCTTGCATGCCTGCACGCAACAGATCAGGTTTG	Rev primer region B – PhoA2
KO2064_3249_AF	AGCAGGGTTATGCAGCGGAAAAATTCGGGGACAATGCCAGCGATGACATC	For primer region A – PhoA1
KO2064_3249_AR	CGGAACTGCTGTATGGATGTGTATTCCTGCGCC	Rev primer region A – PhoA1
KO2064_3249_BF	GCAGGAATACACATCCATACAGCAGTTCCGGTTC	For primer region B – PhoA1
KO2064_3249_BR	CTATGACCATGATTACGCCAAGCTTGCATGCTTTCCATCTGCCCAAAC	Rev primer region B – PhoA1
KO2064_3250_AF	GCAGCGGAAAAATTCGGGGGATCCTGGATGCGGTTGACAAAGC	For primer region A – SusCD2
KO2064_3250_AR	CAAGACCAGCACGTTTTGTCTAATGCCTGCTC	Rev primer region A – SusCD2
KO2064_3250_BF	GCATTAGACAAAACGTGCTGGTCTTGGAGATC	For primer region B – SusCD2
KO2064_3250_BR	ATTACGCCAAGCTTGCATGCCTGCAGGCCGGAGTAGCATCTGTAATATTTC	Rev primer region B – SusCD2
KO2064_2478_AF	ATTACGCCAAGCTTGCATGCCTGCAGCCCTGCAGATATGATGG	For primer region A – PhoX
KO2064_2478_AR	GTATGAGGGTGAATGTGAAGTAAAATCGATGCTTG	Rev primer region A – PhoX
KO2064_2478_BF	ATTTTACTTCACATTCACCCTCATACCTGGC	For primer region B – PhoX
KO2064_2478_BR	GCAGCGGAAAAATTCGGGGGATCCTCGGTTTTGGAATAACTTGGC	Rev primer region B – PhoX
KO2064_0023_AF	GCAGCGGAAAAATTCGGGGGATCCTAAGAGCTCATATCGACGG	For primer region A – PafA_Fj
KO2064_0023_AR	CCGGAACATGGGTGGACGTTGTTGTGCACTC	Rev primer region A – PafA_Fj
KO2064_0023_BF	ACAACAACGTCCACCCATGTTCCGGCTATTTTC	For primer region B – PafA_Fj
KO2064_0023_BR	ATTACGCCAAGCTTGCATGCCTGCACTGAAGTTGCCGGTCGTATG	Rev primer region B – PafA_Fj
KO2064_0074_AF	GTTATGCAGCGGAAAAATTCGGGGGATCCTAGAATACGAACCGGAAGC	For primer region A – 0074
KO2064_0074_AR	TGAAGGTCTACAAATTCCATTTGAATCCTGACC	Rev primer region A – 0074
KO2064_0074_BF	CAGGATTCAAATGGAATTTGTAGACCTTCACGCG	For primer region B – 0074
KO2064_0074_BR	CCATGATTACGCCAAGCTTGCATGCCTGCATACCGGGGCTTTTGTTGG	Rev primer region B – 0074

Compl_pcP0023F	TGCAGCGGAAAAATTCGGGGTGTGGGAGAGTAT GTCGTC	For primer for PafA_Fj complementation – 5' end of promoter
Compl_pcP0023R	AGTCGGCGGCCGCTCTAGAGTTATTTCTTATTAT CTAAAACTTCAGTCATAAC	Rev primer for PafA_Fj complementation – 3' end of fj_0023

Table S2. List of strains used and generated in this study:

**Strain**	**Description** *fjoh_0023*	**Reference**
F. johnsoniae DSM2064	Wild type strain	DSMZ
P. putida BIRD-1	Wild type strain	(*3*)
Polaribacter sp. MED152	Wild type marine *Flavobacteriia* (*Bacteroidetes*)	(*4*)
Gramella forsetii KT0803	Wild type marine *Flavobacteriia* (*Bacteroidetes*)	(*5*)
Formosa agariphila KMM 3901	Wild type marine *Flavobacteriia* (*Bacteroidetes*)	(*6*)
Algoriphagus machipongonensis PR1	Wild type marine *Cytophagia* (*Bacteroidetes*)	(*7*)
Ruegeria pomeroyi DSS-3	Wild type marine *Rhodobacteraceae* (*Alphaproteobacteria*)	DSMZ
Dinoroseobacter shibae DFL-12	Wild type marine *Rhodobacteraceae* (*Alphaproteobacteria*)	DSMZ
Roseobacter denitrificans OCh 114	Wild type marine *Rhodobacteraceae* (*Alphaproteobacteria*)	DSMZ
Δ*phoX::Gm*	Mutant strain fo *P. putida* with *PPUBIRD1_1093 phoX* mutated	(*8*)
Δ*phoX::Gm* +pBX:*phoX*^{BIRD}	Δ*phoX::Gm* complemented with pBBR1MCS-km containing the *phoX* promoter (+pBX) and its native *phoX*	(*8*)
Δ*phoX::Gm* +pBX:*phoA*^{Ec}	Δ*phoX::Gm* complemented with +pBX containing the *E. coli phoA*	This study
Δ*phoX::Gm* +pBX:*phoA1*^{Fj}	Δ*phoX::Gm* complemented with +pBX containing *fjoh_3249* (*phoA1*)	This study
Δ*phoX::Gm* +pBX: *phoA2*^{Fj}	Δ*phoX::Gm* complemented with +pBX containing *fjoh_3187* (*phoA2*)	This study
Δ*phoX::Gm* +pBX: *phoX*^{Fj}	*phoX::Gm* complemented with +pBX containing *fjoh_2478* (*phoX*)	This study
Δ*phoX::Gm* +pBX: *pafA*^{Fj}	*phoX::Gm* complemented with +pBX containing *fjoh_0023* (*pafA*)	This study
phoX::Gm +pBX: *pafA*^{Fj}	Δ*phoX::Gm* complemented with +pBX containing *Cpin_0724* (*pafA1*)	This study
Δ*phoX::Gm* +pBX: *pafA*^{Fj}	Δ*phoX::Gm* complemented with +pBX containing *Cpin_1665* (*pafA2*)	This study
Δ*pafA*	*F. johnsoniae* with *fjoh_0023* (*pafA*) mutated	This study
Δ*phoX*^{Fj}	*F. johnsoniae* with *fjoh_2478* (*pafA*) mutated	This study
Δ*0074*	*F. johnsoniae* with *fjoh_0074* (*pafA*) mutated	This study
Δ*phoA1*:Δ*phoA2*	*F. johnsoniae* with *fjoh_3187* (*phoA2*) *and fjoh_3249* (*phoA2*) mutated	This study
Quad (Δ*phoA1*:Δ*phoA2*: Δ*phoX*^{Fj}:Δ*0074*)	*F. johnsoniae* with *fjoh_0074, fjoh_2478, fjoh_3187* (*phoA2*) *and fjoh_3249* (*phoA2*) mutated	This study
M5 (Δ*phoA1*:Δ*phoA2*: Δ*phoX*^{Fj}:Δ*0074*: Δ*pafA*)	*F. johnsoniae* with *fjoh_0023, fjoh_0074, fjoh_2478, fjoh_3187* (*phoA2*) *and fjoh_3249* (*phoA2*) mutated	This study
M5 +pCP:*pafA*	M5 +pCP11 containing *fjoh_0023* and its native promoter	This study

Table S3. Differences in transcript abundance of the four PMEs across the Pacific Ocean and South Atlantic Ocean combined (see Fig. S7.). Statistical significance was determined by Kruskal-Wallis chi-squared test, followed by Holm's corrected Dunn's test.

Comparison	Z	P.unadj	P.adj
PafA - PhoA	-6.599	<0.001	<0.001
PafA - PhoD	-3.210	0.001	0.004
PafA - PhoX	-4.281989	<0.001	0.003
PhoA - PhoD	3.390	<0.001	0.003
PhoA - PhoX	2.317	0.025	0.041
PhoD – PhoX	-1.073	0.283	0.283

Table S4. Search-algorithm comparison of *phoX* and *phoA* gene abundance in the TARA Oceans dataset. For BLASTP searches, the same query sequences and search parameters as Sebastien et al. (2009) were used.

Phosphatase	No. of hits BLASTP	No. of hits hmmer	Abundance value BLASTP	Abundance value hmmer
phoA	51	947	1020	26114
phoX	409	687	8951	17135

All searches were performed using a stringency e^{-60}.

Supplementary Figures

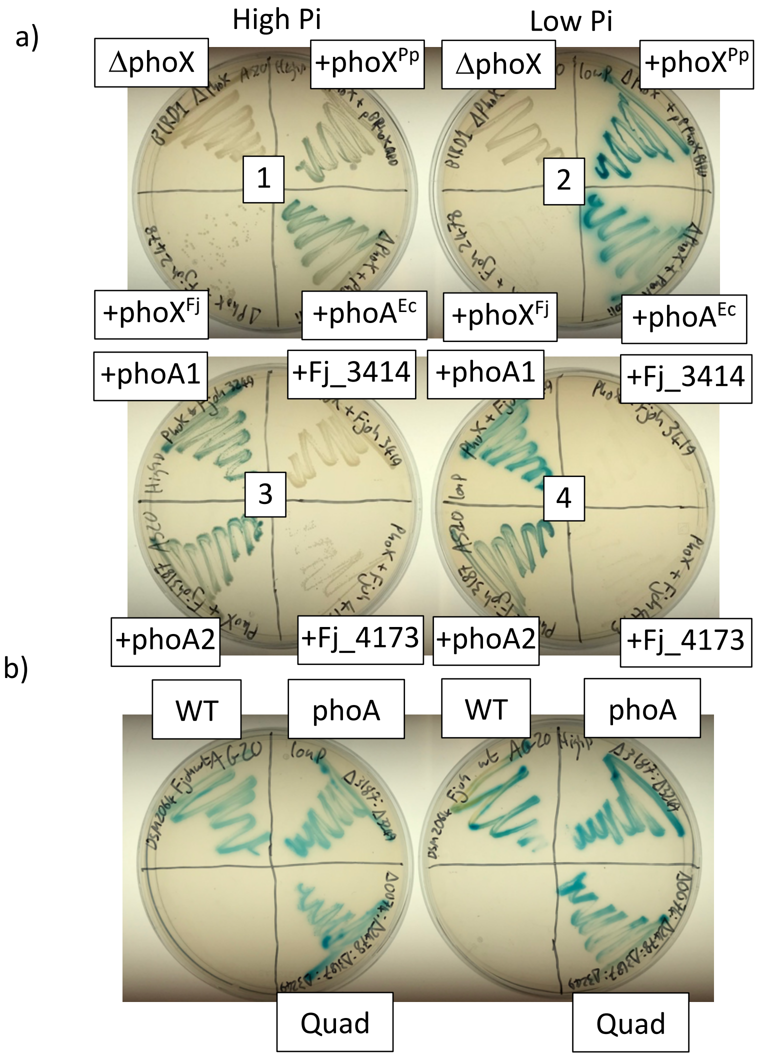

Figure S1. Qualitative plate assay for phosphomonoesterase acitvity. (a) Alkaline phosphatase plate assay using the *Pseudomonas putida* BIRD-1 *ΔphoX* mutant (top left, plates 1 & 2). 5-Bromo-4-chloro-3-indolyl phosphate (BCIP), more commonly known as XP, was used as the substrate. The *phoX* mutant exhibited zero activity indicated by a lack of blue colour which is created when XP is cleaved. Complementation with the native *P. putida* BIRD-1 (+phoX^{Pp}) restored the wild type phenotype. Heterologous expression of the two PhoA-like homologs (Fjoh_3187, bottom left P3 & 4 and Fjoh_3249, top left P3 & 4), also restored APase activity confirming their function. Neither Fjoh_3414 nor Fjoh_4173 restored any phenotype. Interestingly, Fjoh_2478, the PhoX-like homolog, did not restore the phenotype. However, growth in this complemented mutant was inhibited which suggests that expression and subsequent export of the lipoprotein may be affected. Plates were left overnight at 30°C. **(b)** Alkaline phosphatase plate assay using the various *F. johnsoniae* APase mutants. Both the double *phoA* mutant and the quadruple mutant still displayed observable APase activity under Pi replete and Pi deplete growth conditions (top left, plates 1 & 2). 5-Bromo-4-chloro-3-indolyl phosphate (BCIP), more commonly known as XP, was used as the substrate. Abbreviations: WT, wild type; *phoA*, *ΔphoA1: ΔphoA2;* Quad, *ΔphoA1:ΔphoA2:ΔphoX:Δfjoh_0074*.

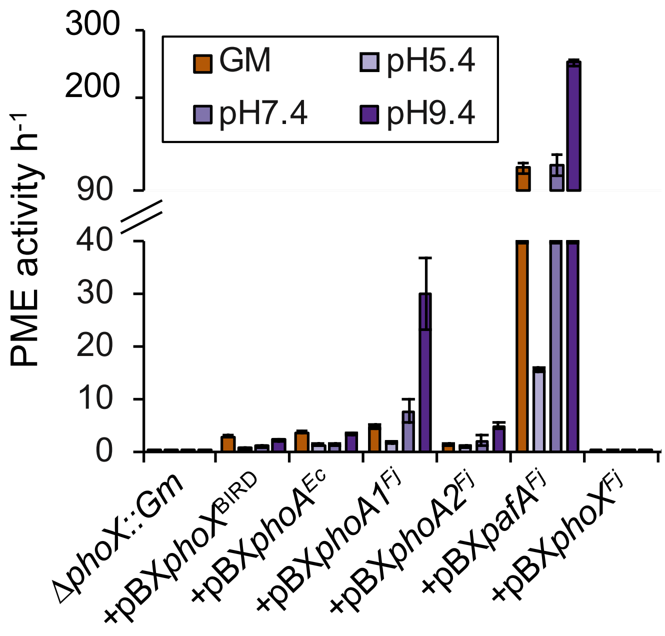

Figure S2. Phosphomonoesterase (PME) activity of a *Pseudomonas putida* Δ*phoX* mutant complemented with PMEs from *P. putida* BIRD (+pBX*phoX*^{BIRD}), *Escherichia coli* (+pBX*phoA*^{Ec}), and *F. johnsoniae* (+pBX*pafA*^{Fj}, +pBX*phoA1*^{Fj}, +pBX*phoA2*^{Fj}, +pBX*phoX*^{Fj}) was recorded in cell cultures grown overnight (n=3) in minimal Pi-deplete medium. PME activity was obtained through addition of the artificial substrate *para*-nitrophenyl phosphate (10 mM) under two conditions: the original growth medium (GM) or by resuspending cells in a buffer adjusted to pH 9.4. Values presented are the mean of biological triplicates and error bars denote standard deviation.

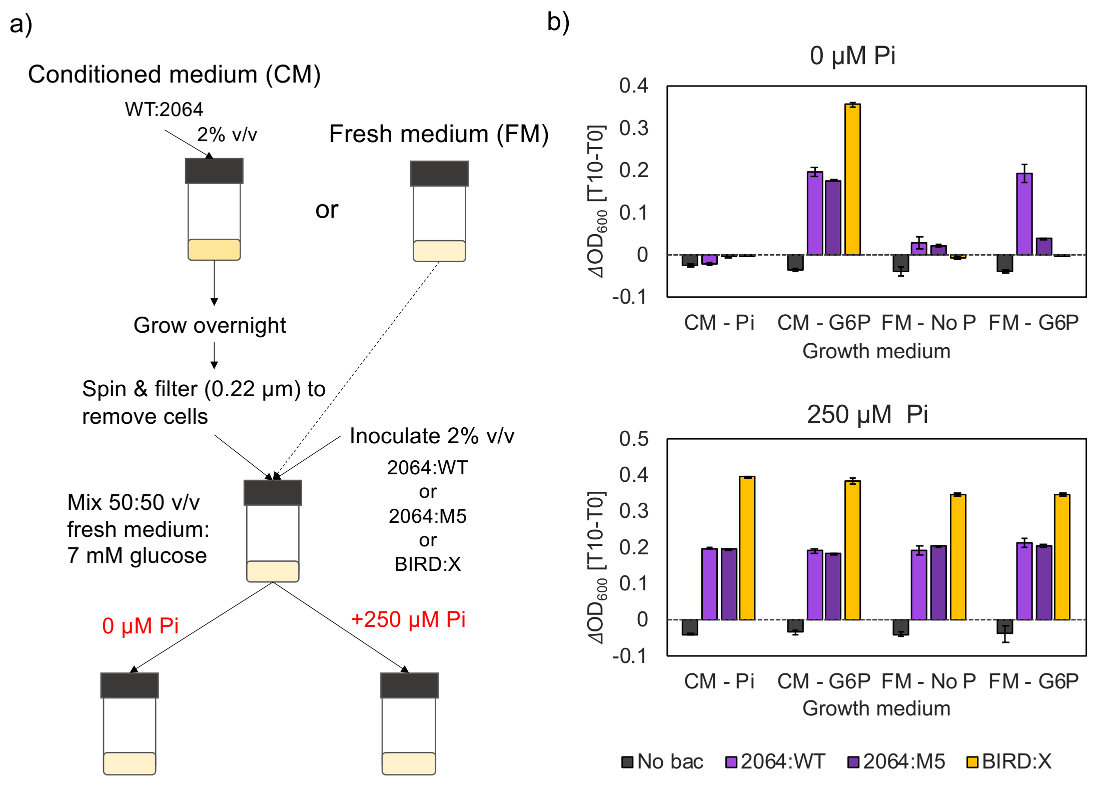

Figure S3. Co-culture growth experiments using organophosphorus growth substrates as the sole P source. (a) Schematic for the generation of conditioned medium (CM) and screening for the accumulation of mineralised phosphate in the growth medium. Briefly, this was achieved by first growing the wild type in minimal medium supplemented with glucose 6-phosphate (2mM) and glucose (7mM), C:P 21:1, which should facilitate accumulation of Pi in response to continual Pi-independent mineralisation by PafA. We also established a control treatment containing glucose (7 mM) and Pi (50 µM) that would create Pi-deplete growth conditions (C:P, 840:1) and thus cause complete consumption of exogenous Pi. After overnight growth, cells were removed via filtration (0.22 µm) and either CM was individually mixed (50:50% v/v) with fresh medium (FM) containing 7 mM glucose and no added P source. Each cell-free growth medium was then either inoculated with the wild type, the quintuple PME mutant or the *P. putida* PME null mutant (Δ*phoX::Gm-BIRD-1*). Control treatments using only fresh medium supplemented with either glucose 6-phosphate or Pi were also established. An additional positive control with 250 µM Pi supplemented to all growth condition-strain combinations was also included. **(b)** Growth of either wild type *F. johnsoniae* (2064: WT), the quintuple PME mutant (2064:M5) or Δ*phoX:Gm-BIRD-1* (BIRD: X) on CM (spent mixed 50:50 with FM) or 100% FM. Cultures with no added bacterial inoculant had no growth, confirming cells were successfully removed from the initial conditioned medium. All positive control cultures supplemented with 250 µM Pi all grew for both fresh medium and conditioned medium treatments (bottom panel). For fresh medium cultures, all strains failed to grow in the absence of Pi and only wild type DSM2064 grew in the presence of glucose 6-phosphate (G6P) whilst the quintuple PME mutant or the *P. putida* mutant did not. No growth was observed for any strain using conditioned medium originally containing 25 µM Pi whilst all three strains grew in conditioned medium originally containing 2mM glucose 6-phosphate (Top panel). Together, these data clearly demonstrate mineralisation of organophosphorus independently of cellular P requirements and production of bioavailable Pi for other organisms. Values represent ΔOD_{600} of cultures (T10 h – T0 h) and are the mean of triplicate cultures. Error bars denote standard deviation.

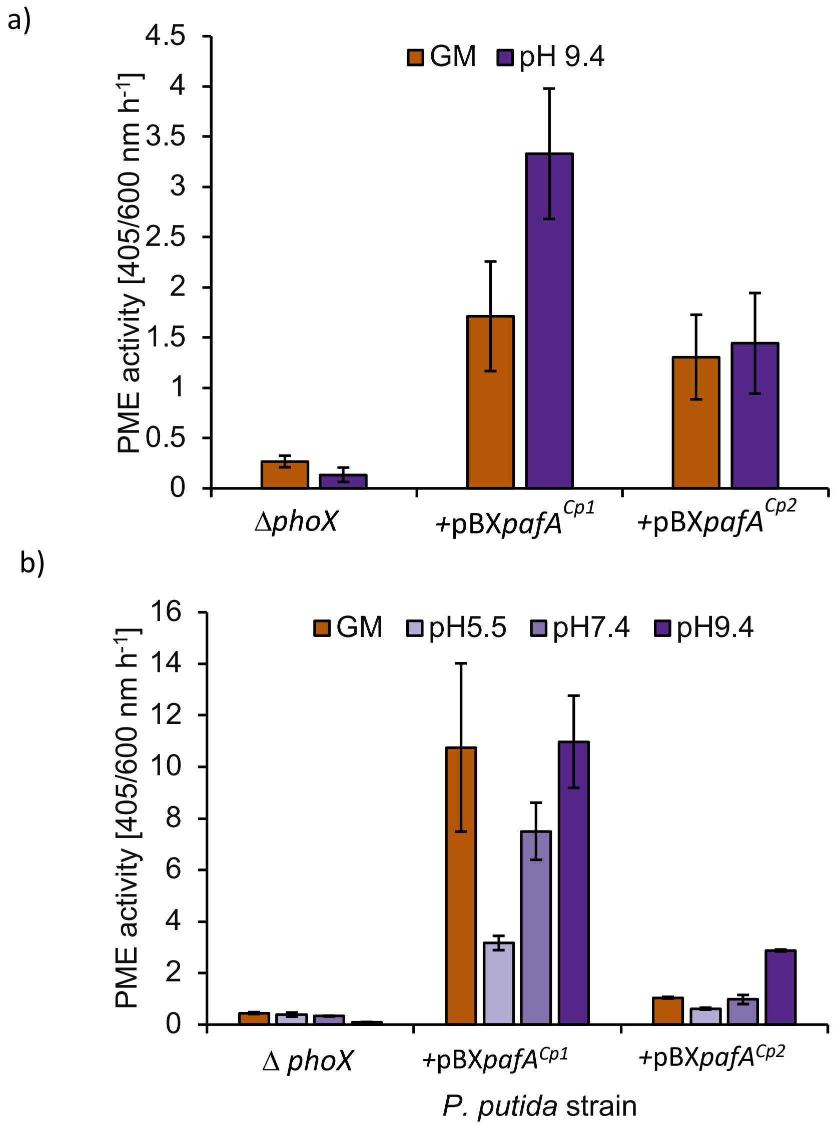

Figure S4. Heterologous phosphomonoesterase (PME) activity in the *P. putida* null mutant. Cells expressing two distinct *pafA* homologs found in the genome of *Chitinophaga pinensis* (+pBX*pafA1*^{Cp} +pBX*pafA2*^{Cp}) were grown overnight in complex medium (**a**) or minimal medium **(b)** established phosphate-deplete (Low Pi) growth conditions. PME activity was obtained through addition of the artificial substrate *para*-nitrophenyl phosphate (10mM). Values presented are the mean of biological triplicates and error bars denote standard deviation. Abbreviations: GM, growth medium.

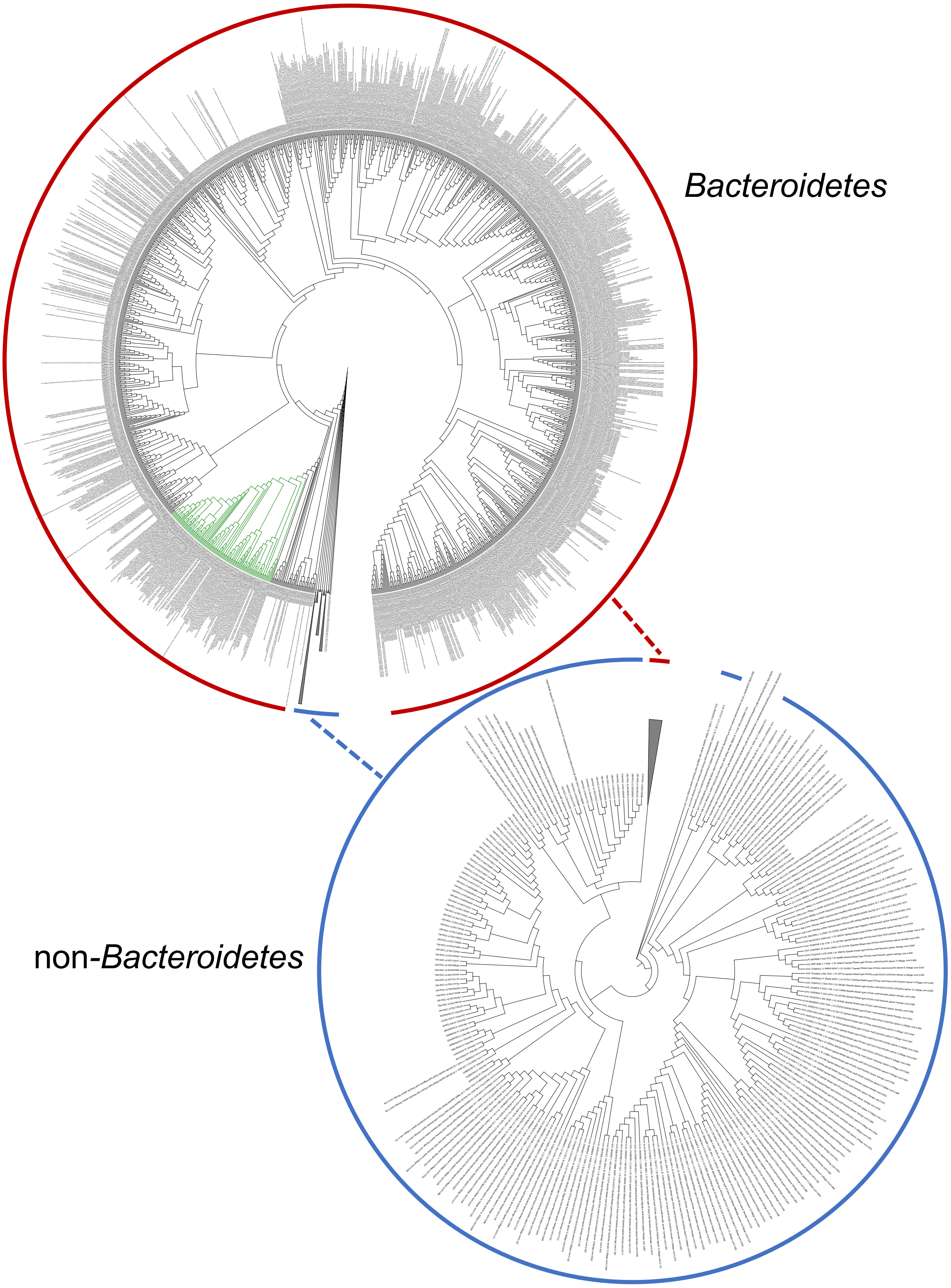

Figure S5. Phylogeny of PafA in genome sequenced isolates and environmental metagenomes. The corresponding ORF_IDs from Fig. 5. are given next to each leaf. The non-*Bacteroidetes* ORF_IDs from collapsed branches in Fig. 5 are presented in the bottom panel. Amino acid sequences are given in SI dataset 1. Branches corresponding to *Flavobacterium* sequences are coloured green (top panel).

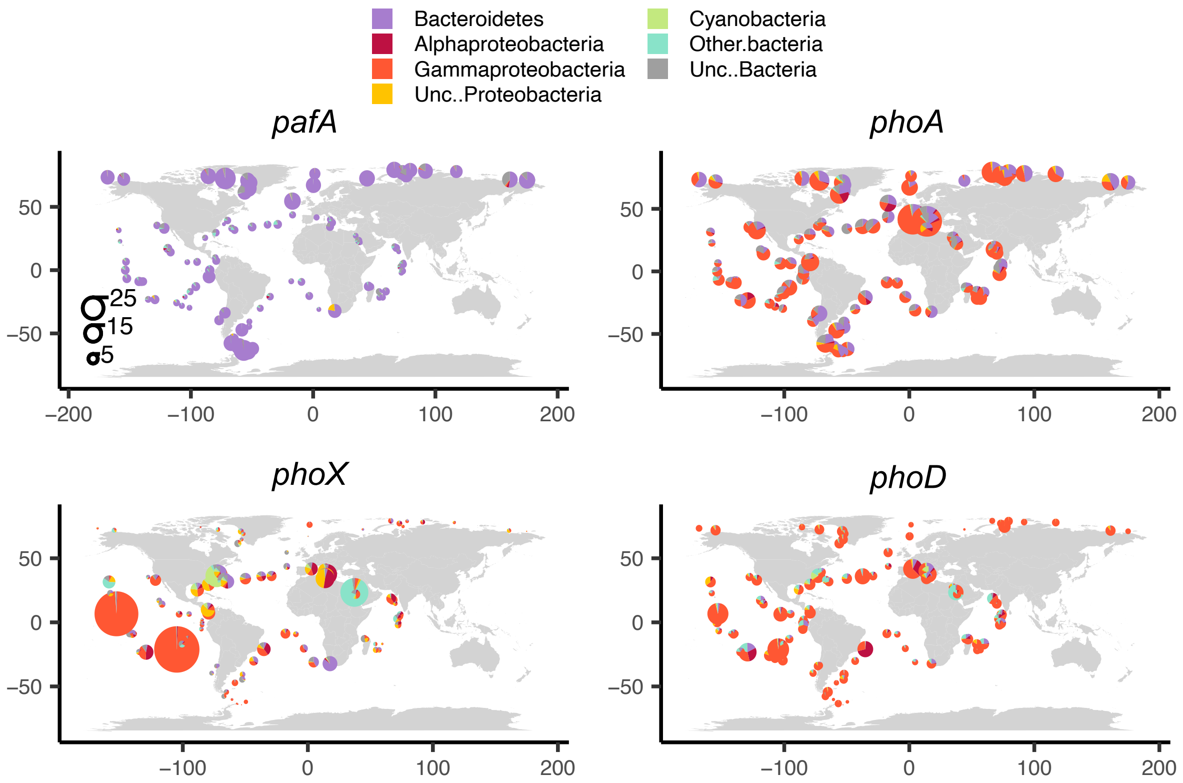

Figure S6. Transcript abundance of the four alkaline PMEs in the global ocean based on the TARA Oceans dataset. The area of each pie chart represents the normalised transcript abundance at each sampling site (SI Dataset 2), expressed as the % reads relative to the average expression of 10 housekeeping genes (see legend for scaling), and the contributing taxa. Note that some sites recorded here are omitted from Fig. S10 due to no data on the standing stock Pi concentration.

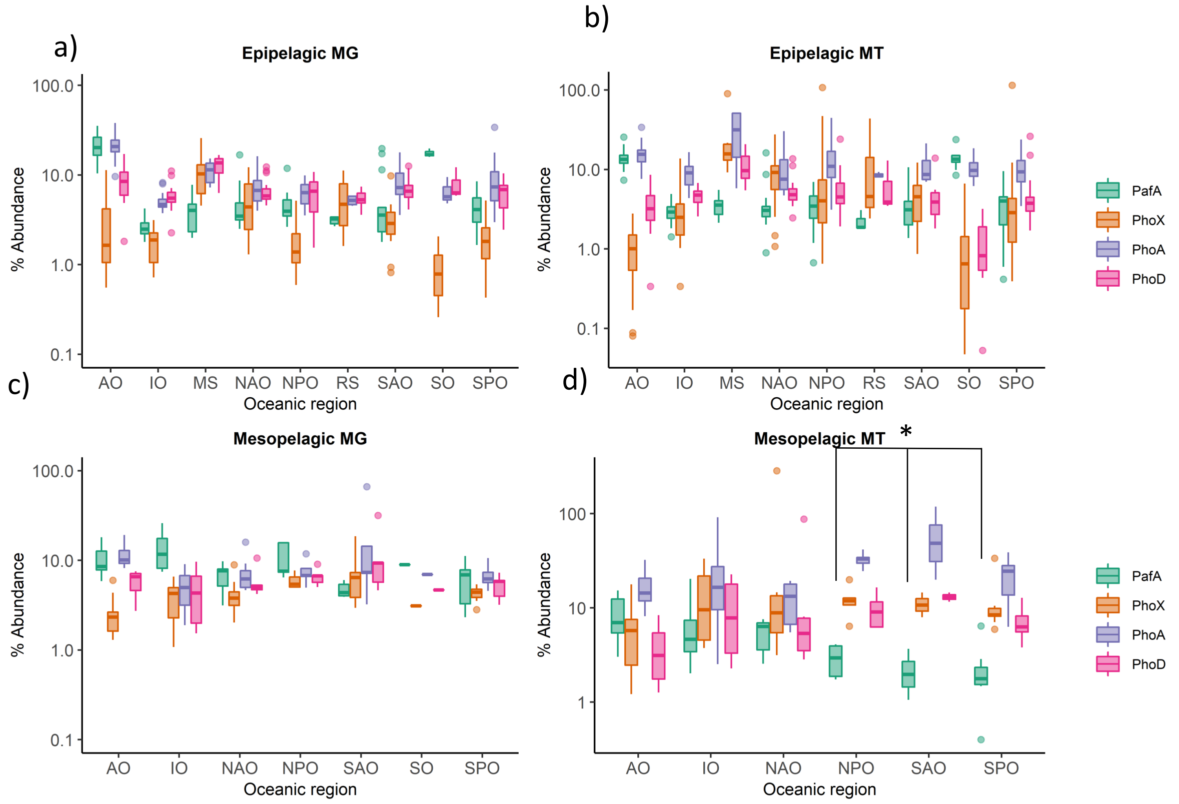

Figure S7. Distribution and expression of phosphatase genes across specific regions of the global ocean. Abundance (Log_{10} abundance [gene or transcript] relative to the median abundance [gene or transcript] of 10 single copy core genes) of *pafA*, *phoX*, phoA, *phoD* in marine epipelagic (**a, b**) and mesopelagic (**c, d**) waters, split by metagenome (MG) (**a, c**) and metatranscriptome (**b, d**). Data are represented as boxplots, where the middle line is the median and the upper and lower hinges correspond to the first and third quartiles. The upper whisker extends from the upper hinge to the largest value that is no more than 1.5×IQR (inter-quartile range) from the upper hinge, and the lower whisker extends from the lower hinge to the smallest value that is no further than 1.5×IQR from the lower hinge. Data beyond the ends of the whiskers are outlier points that are plotted individually.

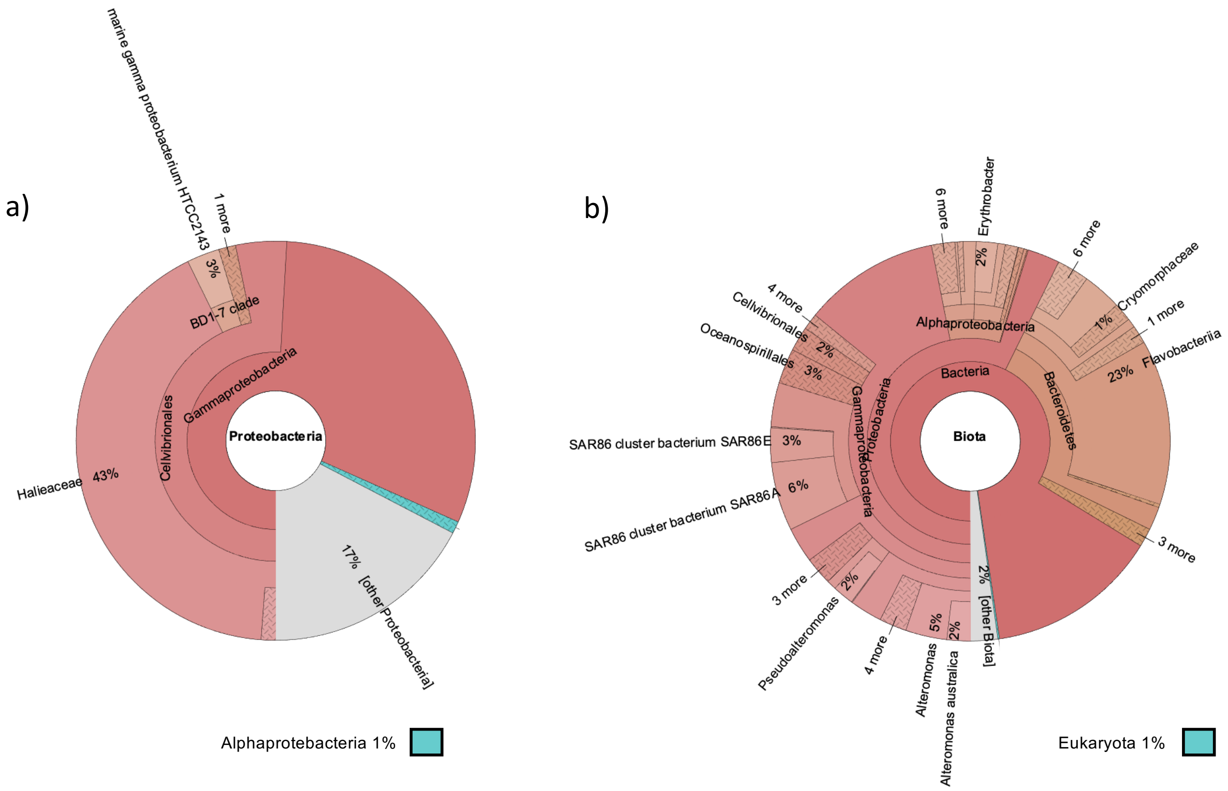

Figure S8. Taxonomic classification of PhoA homologs retrieved from the global ocean. The TARA Oceans OM-RGCv2+G metagenome dataset was scrutinised using either BLASTP (**a**) or hmmer (**b**) search algorithms. Both stringency values were set at e^{-60}.

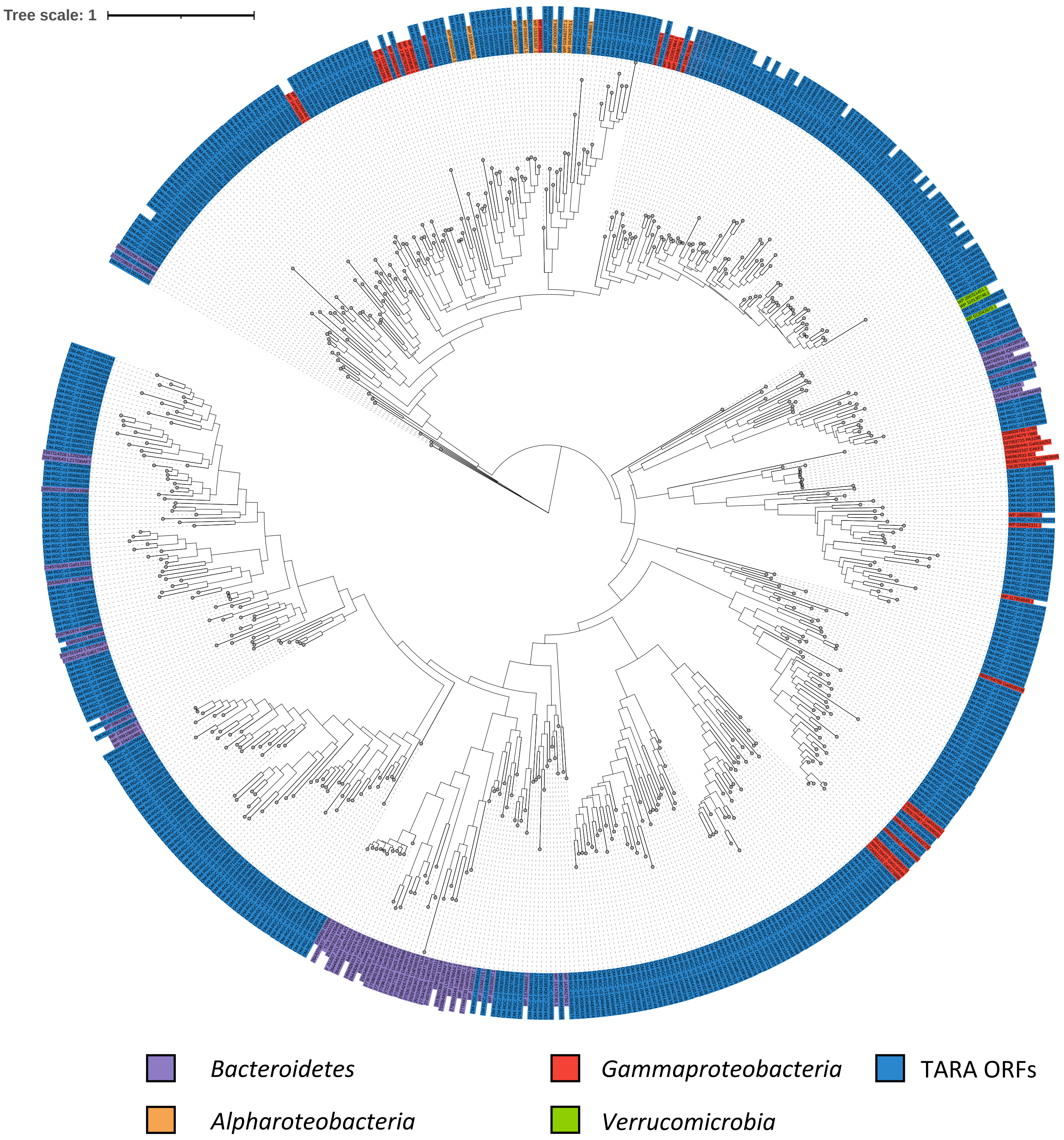

Figure S9. Phylogeny of PhoA homologs retrieved from the TARA oceans dataset. Tree topology and branch lengths were calculated by maximum likelihood using the Blosum62+F+G4 model of evolution for amino acid sequences based on 900 sites in IQ-TREE software. A consensus tree was generated using 1000 bootstraps. TARA ORFs are coloured navy blue. Sequences retrieved from isolate genomes (IMG gene numbers or NCBI accessions provided) were also included and colours represent taxonomy (see legend).

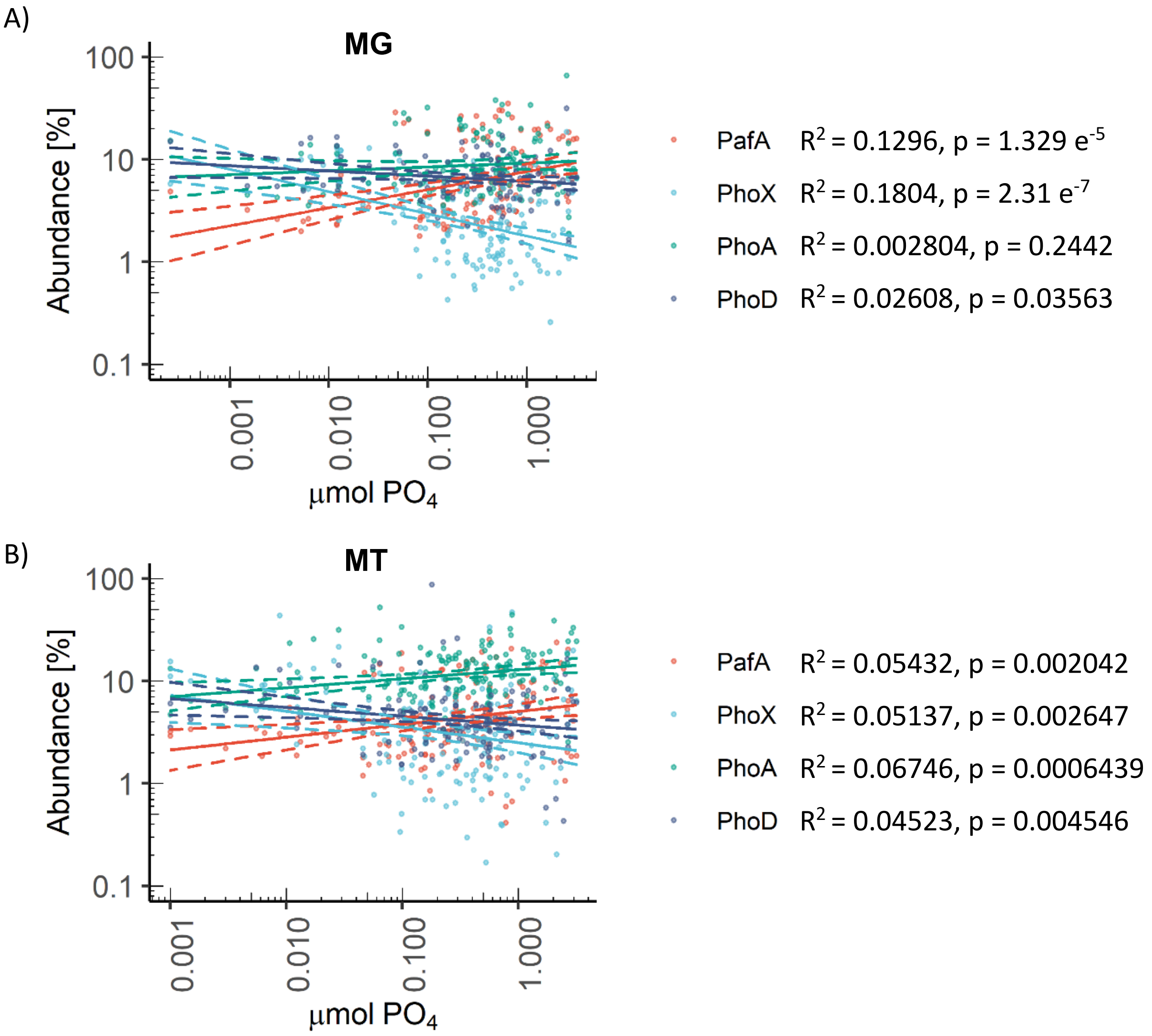

Figure S10. Relationship between gene and transcript abundance of *pafA*, *phoX, phoA*, *phoD* and standing stock phosphate concentrations in the global ocean. Phosphatase abundance, analysed by linear regression of Log$_{10}$ gene abundance and standing stock phosphate (PO_4) concentrations at all sites in the MG (**a**) or MT **(b)** for all sites with epipelagic and mesopelagic merged. 95% confidence intervals are shown by dashed lines. The relationship between the standing stock Pi concentration and degradation marker abundance, analysed by linear regression of Log$_{10}$ PO_4 concentration and Log$_{10}$ gene/transcript abundance. Regression statistics are presented in the figure.

References

1. Y. Zhu, F. Thomas, R. Larocque, N. Li, D. Duffieux, L. Cladière, F. Souchaud, G. Michel, M. J. McBride, Genetic analyses unravel the crucial role of a horizontally acquired alginate lyase for brown algal biomass degradation by *Zobellia galactanivorans*. *Environmental Microbiology* **19**, 2164-2181 (2017).
2. M. E. Kovach, P. H. Elzer, D. Steven Hill, G. T. Robertson, M. A. Farris, R. M. Roop Ii, K. M. Peterson, Four new derivatives of the broad-host-range cloning vector pBBR1MCS, carrying different antibiotic-resistance cassettes. *Gene* **166**, 175-176 (1995).
3. A. Roca, P. Pizarro-Tobías, Z. Udaondo, M. Fernández, M. A. Matilla, M. A. Molina-Henares, L. Molina, A. Segura, E. Duque, J.-L. Ramos, Analysis of the plant growth-promoting properties encoded by the genome of the rhizobacterium *Pseudomonas putida* BIRD-1. *Environmental Microbiology* **15**, 780-794 (2013).
4. J. M. González, B. Fernández-Gómez, A. Fernàndez-Guerra, L. Gómez-Consarnau, O. Sánchez, M. Coll-Lladó, J. del Campo, L. Escudero, R. Rodríguez-Martínez, L. Alonso-Sáez, M. Latasa, I. Paulsen, O. Nedashkovskaya, I. Lekunberri, J. Pinhassi, C. Pedrós-Alió, Genome analysis of the proteorhodopsin-containing marine bacterium *Polaribacter* sp. MED152 (Flavobacteria). *Proceedings of the National Academy of Sciences* USA **105**, 8724 (2008).
5. H. Eilers, J. Pernthaler, J. Peplies, F. O. Glöckner, G. Gerdts, R. Amann, Isolation of novel pelagic bacteria from the German Bight and their seasonal contributions to surface picoplankton. *Applied and Environmental Microbiology* **67**, 5134-5142 (2001).
6. A. J. Mann, R. L. Hahnke, S. Huang, J. Werner, P. Xing, T. Barbeyron, B. Huettel, K. Stüber, R. Reinhardt, J. Harder, F. O. Glöckner, R. I. Amann, H. Teeling, The genome of the alga-associated marine flavobacterium *Formosa agariphila* KMM 3901T reveals a broad potential for degradation of algal polysaccharides. *Applied and Environmental Microbiology* **79**, 6813-6822 (2013).
7. R. A. Alegado, J. D. Grabenstatter, R. Zuzow, A. Morris, S. Y. Huang, R. E. Summons, N. King, *Algoriphagus machipongonensis* sp. nov., co-isolated with a colonial choanoflagellate. *International Journal of Systematic and Evolutionary Microbiology* **63**, 163-168 (2013).
8. I. D. E. A. Lidbury, A. R. J. Murphy, T. D. Fraser, G. D. Bending, A. M. E. Jones, J. D. Moore, A. Goodall, M. Tibbett, J. P. Hammond, D. J. Scanlan, E. M. H. Wellington, Identification of extracellular glycerophosphodiesterases in *Pseudomonas* and their role in soil organic phosphorus remineralisation. *Scientific Reports* **7**, 2179-2179 (2017).
